# Investigating the response of the butyrate production potential to major fibers in dietary intervention studies

**DOI:** 10.1038/s41522-024-00533-5

**Published:** 2024-07-30

**Authors:** Thao Van-Wehle, Marius Vital

**Affiliations:** 1 Institute for Medical Microbiology and Hospital Epidemiology, Hannover Medical School, Hannover, Germany; 2 German Center for Infection Research (DZIF), partner site Hannover-Braunschweig, Hannover, Germany

**Keywords:** Microbiome, Bacteria

## Abstract

Interventions involving dietary fibers are known to benefit host health. A leading contribution of gut microbiota is commonly recognized with production of short chain fatty acids (SCFA) suspected to play a key role. However, the detailed mechanisms are largely unknown, and apart from a well-described bifidogenic effect of some fibers, results for other bacterial taxa are often incongruent between studies. We performed pooled analyses of 16S rRNA gene data derived from intervention studies (n = 14) based on three fibers, namely, inulin-type fructans (ITF), resistant starch (RS), and arabinoxylan-oligosaccharides (AXOS), harmonizing the bioinformatics workflow to reveal taxa stimulated by those substrates, specifically focusing on the SCFA-production potential. The results showed an increased butyrate production potential after ITF (p < 0.05) and RS (p < 0.1) treatment via an increase in bacteria exhibiting the enzyme butyryl-CoA:acetate CoA-transferase (*but*) that was governed by *Faecalibacterium*, *Anaerostipes* (ITF) and *Agathobacter* (RS) respectively. AXOS did not promote an increase in butyrate producers, nor were pathways linked to propionate production stimulated by any intervention. A bifidogenic effect was observed for AXOS and ITF, which was only partly associated with the behavior of *but*-containing bacteria and largely represented a separate response. Low and high *Ruminococcus* abundances pre-intervention for ITF and RS, respectively, promoted an increase in *but*-containing taxa (p < 0.05) upon interventions, whereas initial *Prevotella* abundance was negatively associated with responses of butyrate producers for both fibers. Collectively, our data demonstrate targeted stimulation of specific taxa by individual fibers increasing the potential to synthesize butyrate, where gut microbiota composition pre-intervention strongly controlled outcomes.

## Introduction

Numerous studies have reported the role of gut microbiota in host health and disease, and developing strategies to manipulate dysbiosed microbiota toward a beneficial state is a collective research effort^1^. The focus often lies on the application of dietary fibers, which are non-digestible carbohydrates of more than three monomers that show beneficial physiological effects^2^. For a subset of fibers, the so-called prebiotics, a microbial role was specifically included in their definition as they are “selectively utilized by host microorganisms conferring a health benefit”^3,4^. The most well-known prebiotics are inulin-type fructans (ITF), polymers of fructose linked by ß-2,1 bonds with a terminal glucose, where a bifidogenic effect, that is selective growth of *Bifidobacteria* spp., has been demonstrated several decades ago^5^. While initial procedures targeting bacteria during intervention studies were based on culturing and selective molecular methods, broad-scale applications of next-generation sequencing techniques enabled comprehensive profiling of gut microbiota composition, providing insights on a community-wide level. Consequently, next to *Bifidobacteria* also other members of the microbiota have been found to be targeted by ITF and other fibers in many studies, with largely incongruent results (recently reviewed by Swanson and colleagues that investigated dietary intervention outcomes from studies based on various fibers and found that apart from the bifidogenic effect, results were highly variable between studies and no assertive conclusions were drawn for other taxa)^2^.

There is a broad consensus that dietary fibers act, at least partly, by (selectively) stimulating microbiota growth and activity^2^. However, the detailed mechanisms underlying the associated health benefits are only partially understood^6^. In addition to differences in study design, subject-specific responses, primarily due to distinct microbiota compositions before intervention, have been emphasized to contribute to heterogeneity^7,8^. Given the discrepancies between studies, it is largely unknown which bacterial taxa are selectively stimulated by what substance. In this context, it should be noted that microbe – host interactions are usually not determined by individual taxa, but are governed by microbial functions, where functional redundancy plays a key role^9^. For instance, short chain fatty acids (SCFA) that are microbiota-derived fermentation end products and have been ascribed to gut homeostasis and host health, making their synthesis a primary goal for dietary interventions^1,10^. The main SCFA are acetate, butyrate and propionate; the former is produced by the vast majority of gut bacteria, whereas butyrate and propionate are only formed by certain members that generally form separate groups^11,12^. SCFA act on various targets throughout the body and have been shown to ameliorate various diseases^13,14^. In fact, it is believed that the bifidogenic effect of fiber supplementation is probably not per se causing benefits for the host. Rather, it has been proposed that *Bifidobacteria*-derived lactate fuels the production of butyrate via cross-feeding mechanisms, promoting beneficial effects^15,16^.

While in vitro studies and animal models help to unravel the degradation mechanisms of fibers in detail and to reveal particular physiological properties of the host metabolism^6^, the most relevant data can be derived from human subjects, in particular from dietary intervention studies that focus on the effects of specific fibers^2^. Intervention studies are complex and can vary in many parameters from the initial design and subject characteristics to methods applied for gut microbiota profiling and bioinformatics procedures as well as subsequent data analysis strategies, all contributing to heterogeneity. While study setups and choices of wet-lab procedures cannot be changed retrospectively, approaches for bioinformatics and data analysis are harmonizable^17^. The aim of the present study was to perform a pooled analysis of raw 16S rRNA gene sequencing data derived from placebo-controlled intervention studies by applying uniform bioinformatics procedures, thereby reducing overall variability between studies. We focused on interventions based on common substrates, namely, ITFs and resistant starches (RS); studies applying the more recently used arabinoxylan-oligosaccharides (AXOS) were included as well. Next to infer taxa selectively stimulated by those substrates, we were specifically interested in gaining insights into key functions of microbiota, namely, the potential to produce SCFA, by applying our recently developed procedure to predict the abundances of butyrate and propionate forming pathways from 16S rRNA gene sequencing data^12^.

## Results

In total, 14 studies were finally included in our analyses, with n = 7 for ITF, n = 5 for RS, and n = 2 for AXOS (Figure S1). Intervention designs along with other characteristics are shown in Table 1^8,18–29^. For simplicity, studies involving ITF were not stratified according to chain length; per definition, inulin has at least 10 sugar monomers, whereas shorter molecules are termed fructo-oligosaccharides (FOS). All studies that applied RS were analyzed together irrespective of the RS type; interventions with RS derived from different sources in studies C and K were treated as separate studies. Furthermore, intervention designs differed between studies and data were put into a cross-sectional manner for all studies, subsequently comparing the data of the intervention group with those derived from a placebo control group. The bioinformatics workflow was harmonized for all studies, and linear mixed-effect models (*lm*) that included studies as a random effect (random intercept and random slope; *lmer*) were applied. All results are expressed as changes of the fiber intervention in comparison to the placebo group. In the main Figures results based on relative response data, that is, values post intervention relative to pre-intervention data (RR) are given, whereas those based on changes in relative abundance (RA) are provided as supplementary information. Results are expressed as the estimate from linear models, representing the mean difference between intervention and placebo groups, along with their 95% confidence intervals given in squared brackets.

**Table 1:** Major characteristics of studies included in the pooled analyses

Acrn	Reference	Prebiotic	Dose [g d^-1^]	Subjects	Design	Plc	Int period	16S rRNA
**A**	Healey et al.,^18^	Inulin (chicory root)	16	Healthy adults (n = 20)	Pre vs. plc, cross-over	Mdex	3 weeks	V3-V4
**B**	Sloan et al.,^19^	FOS	14	Healthy adults (n = 37)	Pre vs. plc, parallel	Mdex	1 week	V3-V4
**C**	Baxter et al.,^20^	Inulin (chicory root)	20	Healthy adults (n = 174)	Pre vs. plc, parallel	Asps	2 weeks	V4
**D**	Ford et al.,^21^	Inulin (chicory root)	5,6	Healthy women (n = 26)	Pre vs. plc, cross-over	Mdex	2 weeks	V4
**E**	Hiel et al.,^22^	Inulin (chicory root)	16	Healthy obese adults (n = 106)	Pre vs. plc, parallel	Mdex	3 months	V5-V6
**F**	Li et al.,^23^	Inulin & FOS	10	Patients on dialysis (n = 15)	Pre vs. plc, cross-over	Mdex	12 weeks	V3-V4
**G**	Reimer et al.,^24^	Inulin (chicory root)	3 vs. 7	Healthy adults (n = 25)	Pre vs. plc, cross-over	Whlgr	4 weeks	V3-V4
**C_m, C_p**	Baxter et al.,^20^	RS (maize, potato)	20	Healthy adults (n = 174)	Pre vs. plc, parallel	Asps	2 weeks	V4
**I**	Upadhyaya et al.,^25^	RS4	30% v/v	Patients Metab. Syn. (n = 20)	Pre vs. plc, cross-over	Flour	12 weeks	V4
**J**	Maier et al.,^26^	RS (maize)	48	Patients Insul. Res. (n = 39)	Pre vs. plc, cross-over	L-RS	2 weeks	V4-V6
**K_m, K_p, K_t**	Deehan et al.,^27^	RS4 (maize, potato, tapioca)	10, 20, 35, 50 (w-increase)	Healthy adults (n = 40)	Pre vs. plc, parallel	Corn Starch	4 weeks	V5-V6
**L**	Kemp et al.,^28^	RS2	16	Patients H-dialysis (n = 20)	Pre vs. plc, parallel	Flour	4 weeks	V4-V5
**M**	Chung et al.,^8^	AXOS	15	Healthy adults > 60 (n = 21)	Pre vs. plc, cross-over	Mdex	10 days	V1-V2
**N**	Mueller et al.,^29^	AXOS	15	Healthy adults (n = 48)	Pre vs. plc, parallel	Mdex	12 weeks	V4

For studies C and K, RS derived from different sources were treated separately. *Mdex* maltodextrin, *Asps* amylase-accessible corn starch, *Whlgr* wholegrain, *Acrn* acronym.

**Fig. 1:**
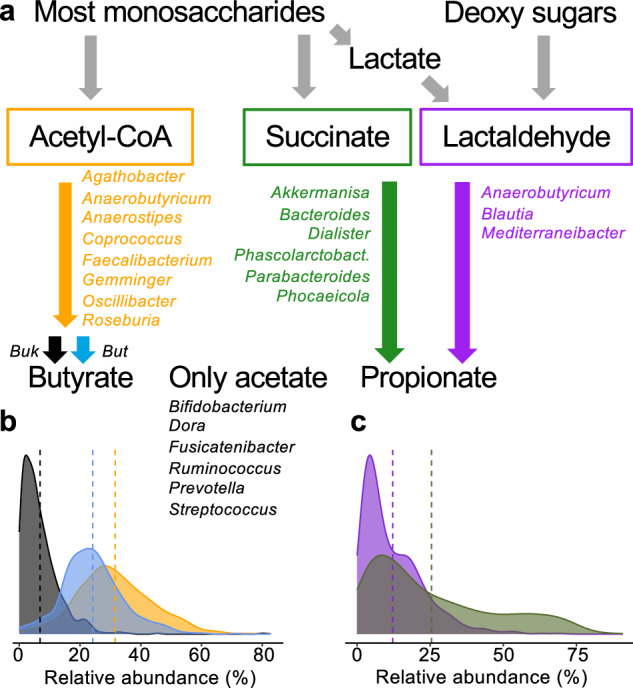
Overview of major butyrate- and propionate-forming pathways and main taxa (on genus level) involved. The Acetyl-CoA pathway (orange) represents the main route for butyrate synthesis and is characterized by two distinct terminal enzymes, namely, butyrate kinase (Buk (black)) and butyryl-CoA:acetate CoA-transferase (But (light blue)); propionate is mainly formed via the Succinate pathway (green) and the Propandiol pathway (Pdiol) (panel **a**). Most abundant taxa that do not form either SCFA are given as well. In panels **b** and **c** the relative abundances of predicted pathways pre-intervention (all studies together) are given.

Our focus was on investigating the effect of fibers on the SCFA-producing potential of gut microbiota. Figure 1a provides an overview of the major pathways leading to butyrate and propionate synthesis, along with the main taxa involved. The butyrate synthesizing acetyl-CoA pathway (AcCoA) is fed by carbohydrates and involves many different taxa, mainly *Firmicutes*. The final step involves two different routes, either via acetate kinase (*buk*) or via butyryl-CoA:acetate CoA-transferase (*but*), which requires acetate as a co-substrate. For propionate, two main pathways exist: the succinate (Suc) pathway linked to major members of gut microbiota belonging to *Bacteroidetes* and some minor contributors of *Firmicutes*, and the propanediol (Pdiol) pathway, which primarily consists of *Firmicutes* taxa. The most abundant genera producing primarily acetate (and lactate) as fermentation end products were also included. The relative pathway abundances for all studies before intervention are shown in Figs. 1b and 1c. On average, 31.57% ± 12.41 of bacteria were predicted to exhibit the AcCoA pathway, with 24.17% ± 10.11 and 6.84% ± 6.48 carrying *but* and *buk*, respectively. The relative abundances of propionate pathways were 25.42% ± 21.17 (Suc) and 12.15% ± 10.40 (Pdiol). The relative abundances of butyrate pathways were in a similar range for all studies, except for Study B, which showed higher values, whereas those for propionate pathways varied considerably between studies (Supplementary Figure 2).

### Intervention outcomes for studies applying ITF

**Fig. 2:**
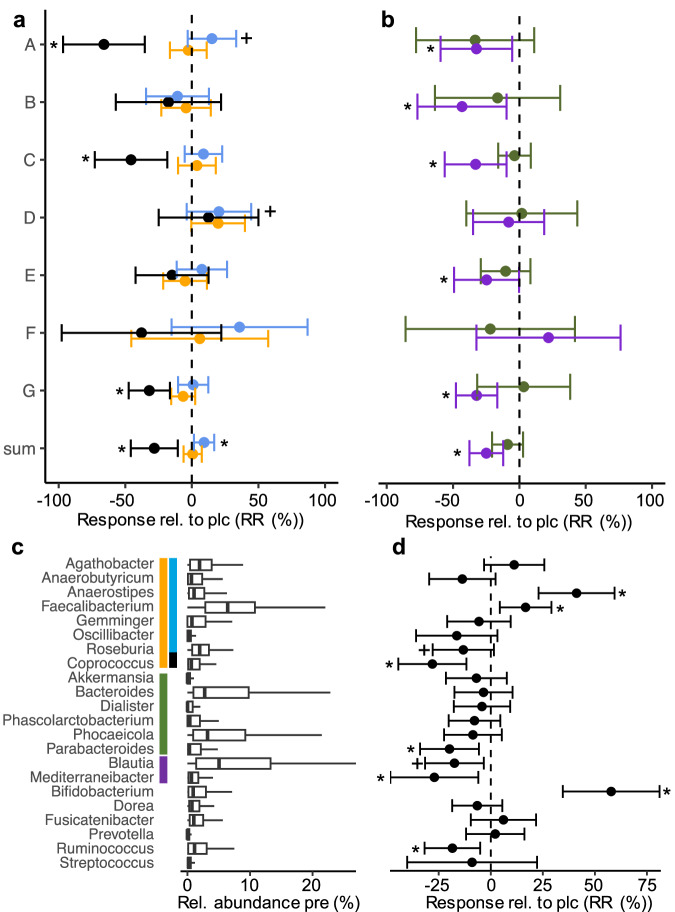
Intervention outcomes based on inulin-type fructans (ITF). Panel **a** shows the response (compared with placebo) based on values relative to pre-intervention (RR) of the main butyrate pathway (orange) along with the two terminal enzymes *but* (light blue) and *buk* (black). Responses of the two propionate-forming pathways (Suc: green and Pdiol: violet) are given in panel **b**. sum: results based on pooled analyses. For detailed characteristics of individual studies see Table 1. Below (panel **c**) relative abundances pre-intervention and responses relative to pre-intervention (compared with placebo) (**d**) of major taxa associated with individual pathways are given. The estimated effect sizes from linear mixed effect models including studies as a random effect (*lmer*) along with their 95% confidence intervals are given. *, +: p < 0.05, p < 0.1 compared with placebo.

Our pooled analysis of the relative response data revealed that ITF treatment did not significantly alter the overall cumulative abundance of AcCoA-exhibiting bacteria (Fig. 2a). However, significant relative increases in bacteria carrying the enzyme *but* (9.15 [1.56, 16.76]; p < 0.05 (*lmer*)) were detected compared to the placebo group, which was mainly driven by *Anaerostipes* (41.19 [22.96, 59.44]; p < 0.01 (*lmer*)) and *Faecalibacterium* (16.71 [4.33, 29.09]; p < 0.05 (*lmer*)), whereas other major taxa from this group were not affected or declined, such as *Roseburia* (-13.19 [-27.92, 1,53]; p < 0.1 (*lmer*)) (Fig. 2c). B*uk*-carrying members declined (-28.20 [-45.84, -10.56]; p < 0.05 (*lmer*)), including its main contributor, *Coprococcus* (-28.05 [-44.43, -11.67]; p < 0.05 (*lmer*)). Analyses of relative abundances gave a similar picture with *Faecalibacterium* and *Anaerostipes* significantly increased in individuals treated with ITF compared with placebo (1.42 [0.48, 2.36]; p < 0.01 (*lmer*), and 1.48 [0.60, 2.36]; p < 0.05 (*lmer*), respectively), along with cumulative *but*-carrying taxa that trended to increase (1.58 [0.14, 3.02]; p < 0.1 (*lmer*)); the overall AcCoA abundance was not affected, whereas *buk*-exhibiting taxa declined (Supplementary Figure 3a and c). Results from individual datasets largely followed the pooled outcome with a positive response of *but*-carrying bacteria (except for study B); however, results of individual studies did not reach statistical significance, only pooled analyses allowed to uncover this effect (Fig. 2a and Supplementary Table 1). Butyrate concentrations measured in the original studies followed the same pattern, increasing in all studies and decreasing in study B; however, similar to microbiota data the values did not reach statistical significance in individual studies (Supplementary Table 2). We found that the major drivers *Anaerostipes* and *Faecalibacterium* were significantly or trended increased in four and two (RR) and five and three (RA) studies, respectively (Supplementary Table 3). Similar results were not reported in the original studies (Supplementary Table 2). Bacteria exhibiting *buk* significantly declined in three studies (RR and RA), with *Coprococcus* declining in four (RR) and three (RA) studies (Supplementary Table 3).

In the case of propionate, the Suc pathway abundance was unaffected, and only a minor member, *Parabacteroides* (RR), responded in decreasing its concentrations (Fig. 2c and Supplementary Figure 3). In contrast, the Pdiol pathway showed a significant negative response to ITF interventions compared with placebo groups in the pooled analysis (-24.91 [-37.65, -12.16]; p < 0.05 (*lmer*)) (Fig. 2b), which was supported by the individual results of five studies (Supplementary Table 1). Fecal propionate concentrations were found to increase in some studies, but not at significant levels (Supplementary Table 2). For its main associated members overall negative response were detected (*Blautia:* -17.45 [-31.55, -3.35]; p < 0.1 (*lmer*), *Mediterraneibacter:* -27.10 -48.23, -5.97]; p < 0.5 (*lmer*) (Fig. 2c), which is in agreement with the individual results of several studies (Supplementary Table 3). When considering the relative abundance changes, the Pdiol pathway was not significantly affected, nor were *Blautia* and *Mediterraneibacter* (Supplementary Figure 3).

A bifidogenic effect was detected with *Bifidobacterium* strongly positively responding to ITF treatment, increasing its relative abundance compared with placebo (57.81 [34.56, 81.07]; p < 0.01 (RR; *lmer*) and 5.19 [2.24, 8.13]; p < 0.01 (RA; *lmer*)) (Fig. 2c), which was supported by the individual results of five studies (Supplementary Table 3); the same five studies also originally reported an increase of this taxon (Supplementary Table 2). *Ruminococcus* (-18.42 [-31.77, -5.07]; p < 0.01 (RR; *lmer*)) declined, whereas other major non-butyrate, non-propionate-synthesizing taxa of gut microbiota did not react differently to ITF compared with the placebo groups.

Pooled analyses (comparing pre- and post-intervention values) on all placebo/control groups, as well as on only those using maltodextrin (n = 7), did not yield significant changes for any pathways and any revealed key taxa (p > 0.1; *lmer;* data not shown).

### Intervention outcomes for studies applying RS

**Fig. 3:**
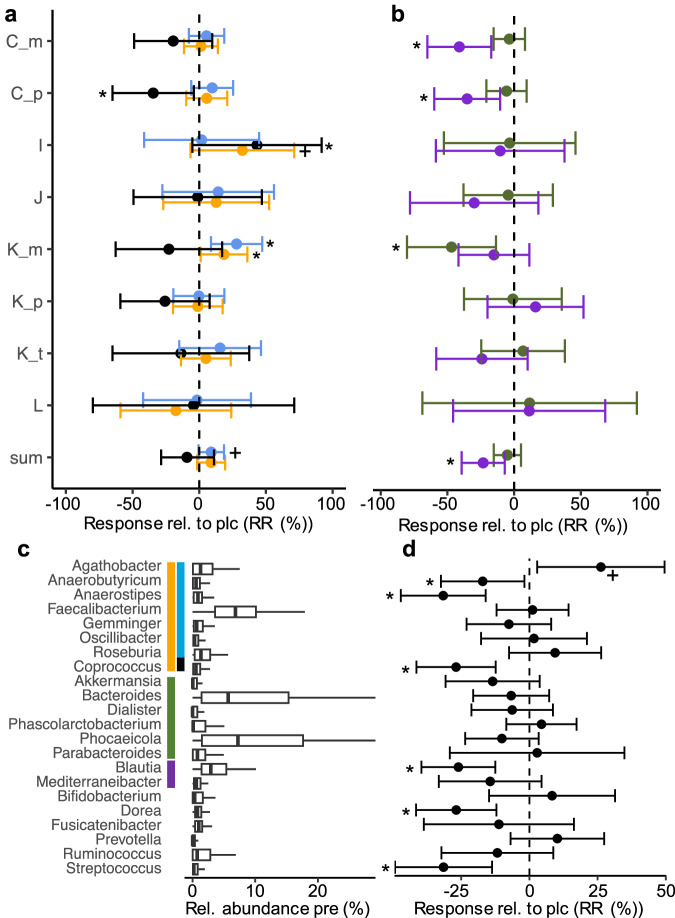
Intervention outcomes based on resistant starches (RS). Panel **a** shows the response (compared with placebo) based on values relative to pre-intervention (RR) of the main butyrate pathway (orange) along with the two terminal enzymes *but* (light blue) and *buk* (black). Responses of the two propionate-forming pathways (Suc: green and route: violet) are given in panel **b**. sum: results based on pooled analyses. For study C and K groups treated with starches from different sources (m: maize; p: potato; t; tapioca) were included as separate studies; for detailed characteristics of individual studies see Table 1. Below (panel **c**) relative abundances pre-intervention and responses relative to pre-intervention (compared with placebo) (**d**) of major taxa associated with individual pathways are given. The estimated effect sizes from linear mixed effect models including studies as a random effect (*lmer*) along with their 95% confidence intervals are given. *, +: p < 0.05, p < 0.1 compared with placebo.

Overall, resistant starches trended to trigger *but*-containing bacteria compared to placebo (8.70 [-0.97, 18.37]; p < 0.1 (*lmer*)) (Fig. 3a), which was in line with a significant increase in measured butyrate in several individual studies (Supplementary Table 2). Bacteria carrying *buk* and the total AcCoA pathway abundance did not change significantly. In contrast to ITF, this increase was primarily governed by *Agathobacter* (26.20 [2.87, 49.54]; p < 0.1 (*lmer*)), whereas major players in the case of ITF, namely, *Faecalibacterium* and *Anaerostipes* were not affected or declined (-31.95 [-47.14, -16.03]; p < 0.01 (*lmer*)), respectively (Fig. 3c). The *buk*-containing *Coprococcus* also declined (-26.88 [-41.38, -12.38]; p < 0.01 (*lmer*)). Based on the relative abundance changes, the same trends were observed; however, the significance for the *but* pathway was slightly above 0.1 (p = 0.101*; lmer*) (Figure S4a); *Agathobacter* increased (1.73 [0.31, 3.16]; p < 0.05 (*lmer*)) (Fig. 3c). Individual datasets supported the pooled results displaying a positive response of *but*-carrying bacteria to RS (except for studies L and K_p (only RR)); however, apart from study K_m, this did not reach significance (Fig. 3a and Supplementary Figure 4a; Supplementary Table 4). Study I was an exception, where the AcCoA pathway trended to increase (p < 0.1 (RA; *lm*)), which was triggered by *buk*-containing bacteria (Fig. 3a), based on relative abundance data (Supplementary Figure 4a). *Agathobacter* was significantly increased in two (RR) and three (RA) studies (Supplementary Table 5). Two original studies have reported an increase in *Eubacterium rectale* (now *Agathobacter rectale*).

In the case of propionate, the Pdiol pathway significantly declined in the analyses based on RR (-23.19 [-39.33, -7.05]; p < 0.05 (*lmer*); Fig. 3b) and RA (-1.06 [-1.80, -0,32]; p < 0.01 (*lmer*); Supplementary Figure 4b), which was governed by *Blautia* (-26.02 [-39.56, -12.49]; p < 0.01 (RR; *lmer*), -0.73 [-1.26, -0.20]; p < 0.05 (RA; *lmer*)) (Fig. 3c). The Suc pathway and its main associated taxa were hardly affected (Fig. 3 and Supplementary Figure 4). The fecal propionate concentrations showed a mixed pattern (Supplementary Table 2).

The concentrations of *Bifidobacterium* increased but did not reach significance (Fig. 3c and Supplementary Figure 4c). None of the original studies have reported an increase in this taxon (Supplementary Table 2). The non-butyrate, non-propionate-synthesizing *Dorea* (-26.82 [-41.54, -12.09]; p < 0.01 (RR; *lmer*)) and *Streptococcus* (-31.47 [-49.26, -13.68]; p < 0.01 (RR; *lmer*), (-1.06 [-1.88, -0.23]; p < 0.05 (RA; *lmer*)) declined upon RS intervention compared to placebo (Fig. 3c and Supplementary Figure 4c).

### Intervention outcomes for studies applying AXOS

**Fig. 4:**
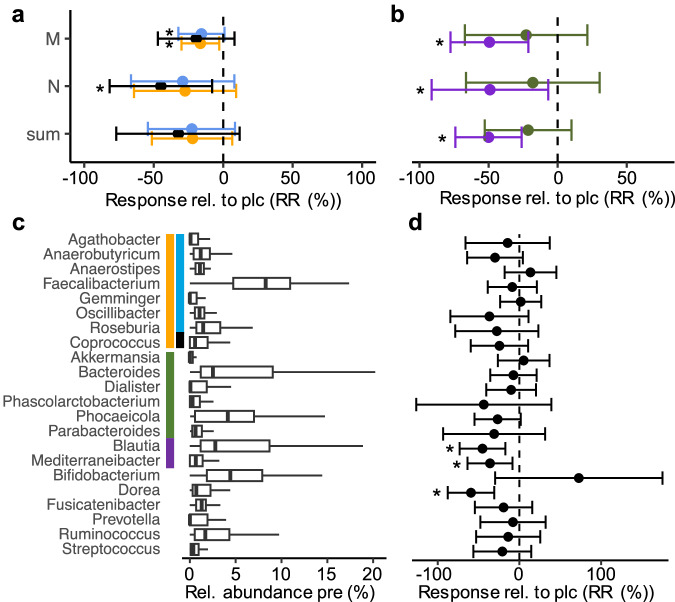
Intervention outcomes based on arabinoxylan-oligosaccharides (AXOS). Panel **a** shows the response (compared with placebo) based on values relative to pre-intervention (RR) of the main butyrate pathway (orange) along with the two terminal enzymes *but* (light blue) and *buk* (black). Responses of the two propionate-forming pathways (Suc: green and Pdiol: violet) are given in panel **b**. sum: results based on pooled analyses. For detailed characteristics of individual studies see Table 1. Below (panel **c**) relative abundances pre-intervention and responses relative to pre-intervention (compared with placebo) (**d**) of major taxa associated with individual pathways are given. The estimated effect sizes from linear mixed effect models including studies as a random effect (*lmer*) along with their 95% confidence intervals are given. *, +: p < 0.05, p < 0.1 compared with placebo.

AXOS treatment had no overall effect on the butyrate-producing potential of gut microbiota (Fig. 4 and Supplementary Figure 5). For study M, both the AcCoA pathway (-16.42 [-29.99, -2.85]; p < 0.05 (RR*; lm*) and -3.63 [-6.60, -0.65]; p < 0.05 (RA*; lm*)) along with *but*-carrying taxa (-15.65 [-32.22, 0.92]; p < 0.1 (RR; *lm*), and -2.60 [-5.18, -0.02]; p < 0.05 (RA*; lm*)) were significantly decreased (respective RA data of study N trended decreased (AcCoA: -6.73 [-14.06, 0.60]; p < 0.1 (*lm*), *but:* -5.42 [-11.42, 0.59]; p < 0.1 (*lm*)) compared to the control groups (Supplementary Table 6). The relative abundance of *Faecalibacterium* decreased (-1.71 [-3.44, -0.03]; p < 0.1 (*lmer*)) (Supplementary Figure 5 and Supplementary Table 7). SCFA measurements in study M suggested a slight, non-significant increase in fecal butyrate levels, whereas the metabolite was unaffected in study N (Supplementary Table 2).

For propionate, the Pdiol pathway responded negatively in the pooled analysis (-49.96 [-74.15, -25.77]; p < 0.01 (*lmer*)) and in both individual studies (Fig. 4 and Supplementary Table 6). The two major taxa linked to the pathway also responded significantly (*Blautia*: -45.19 [-73.23, -17.16], p < 0.01 (*lmer*); *Mediterraneibacter:* -35.96 [-63.46, -8.46], p < 0.05 (*lmer*)) (Fig. 4c; Supplementary Table 7). Based on the pooled analysis, the relative abundance did not change for this pathway or for associated major taxa (Supplementary Figure 5).

AXOS displayed a strong increase in *Bifidobacteria* which was, however, only significant in RA data (10.96 [4.37, 17.55]; p < 0.05 (*lmer*)) and was supported by the results of the two individual studies; for study M, the taxon also increased based on RR (Supplementary Table 7). These results were congruent with those of the original studies (Supplementary Table 2). *Dorea* decreased in the pooled analyses (-59.26 [-87.75, -30.77]; p < 0.01 (RR*; lmer*)) and in both individual studies (RR and RA).

### Bacterial composition pre-intervention influenced intervention outcomes

**Fig. 5:**
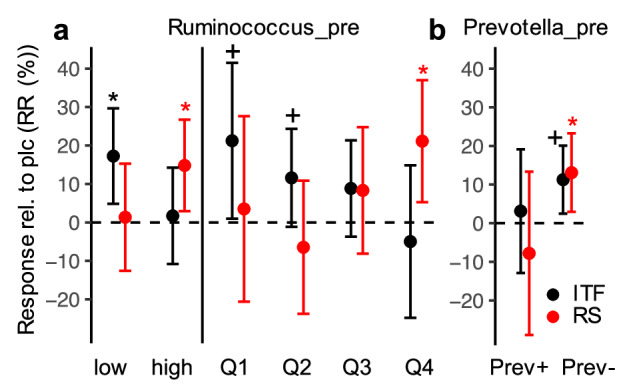
Relative abundances of *Ruminococcus* and *Prevotella* pre-intervention affecting increase of *but*-containing bacteria upon ITF and RS treatment. Panel **a** give results after stratification by *Ruminococcus* abundance based on median abundances (low: below median, high: above median) and quartile abundances (Q1-Q4), whereas panel **b** shows outcomes after stratification for *Prevotella* abundance, where Prev+ refers to samples comprising >= 1% *Prevotella* relative abundance pre-intervention (Prev- represent all other samples). Results display responses of *but*-exhibiting bacteria relative to pre-intervention (RR) compared with placebo. Stratifications were performed for each study individually. The estimated effect sizes from linear mixed effect models including studies as a random effect (*lmer*) along with their 95% confidence intervals are given. *, +: p < 0.05, p < 0.1 compared with placebo.

We investigated whether the bacterial composition pre-intervention influenced the increase in *but*-containing bacteria. We focused on *Prevotella* and *Ruminococcus* which have been reported to play key roles in substrate degradation and represent signature taxa of the so-called enterotypes. Furthermore, associations between the bifidogenic effect and the responses of *but*-containing taxa upon intervention were analyzed. For ITF, lower *Ruminococcus* and *Prevotella* relative abundances pre-intervention triggered a response in *but*-containing bacteria in stratified analyses (Fig. 5). *But*-containing taxa significantly increased compared to the controls (17.25 [4.84, 29.66]; p < 0.05 (*lmer*)) only in the group characterized by initial *Ruminococcus* abundances below the median (Fig. 5a); stratifications based on quartiles underlined the observed effect. In the case of *Prevotella* only the group of subjects exhibiting the taxon abundance below 1% pre-intervention responded (11.28 [2.46, 20.09]; p < 0.1 (*lmer*)) (Fig. 5b). Similar results were obtained for the RA data (Figure S6). The increase in *Bifidobacterium* correlated with the responses of *Anaerobutyricum* (only RR), *Anaerostipes*, and *Faecalibacterium* (only RR), however, no correlation with the cumulative outcome of *but*-containing taxa was observed (Table 2).

**Table 2:** Correlations between responses of *Bifidobacterium* and butyrate producers

	*Bifidobacterium_ITF*	*Bifidobacterium_RS*	*Bifidobacterium_AXOS*
***but (RR)***	n.s.	n.s.	*0.00*
***but (RA)***	n.s.	*0.08*	*0.00*
***Agathobacter (RR)***	n.s.	n.s.	n.s.
***Agathobacter (RA)***	n.s.	n.s.	n.s.
***Anaerostipes (RR)***	0.00	0.00.	n.s.
***Anaerostipes (RA)***	0.02	n.s.	0.08
***Anaerobutyricum (RR)***	0.00	0.00	n.s.
***Anaerobutyricum (RA)***	n.s.	0.00	*0.00*
***Faecalibacterium (RR)***	0.08	n.s.	*0.10*
***Faecalibacterium (RA)***	n.s.	*0.01*	*0.00*

Results (p-values based on linear mixed-effect models (lmer)) for cumulative *but*-containing taxa as well as associated key members revealed in this study are shown. Normal font: positive association; italic font: negative association; *n.s.* not significant, *RR* response relative to pre-intervention, *RA* response based on relative abundance change.

In contrast to ITF, high *Ruminococcus* relative abundances pre-intervention promoted *but*-containing bacteria (14.81 [2.92, 26.71]; p < 0.05 (RR; *lmer*) and 2.44 [-0.10, 4.98]; p < 0.1 (RA; *lmer*)) upon intervention with RS (Supplementary Figure 5a and Supplementary Figure 6a). In line with ITF, low initial *Prevotella* abundance was associated with increased responses in this group (13.09 [2.93, 23.26]; p < 0.05 (RR*; lmer*) and 2.13 [-0.05, 4.32]; p < 0.1 (RA*; lmer*)). While responses of *Bifidobacterium* were correlated with those of *Anaerostipes* (only RR) and *Anaerobutyricum* they trended to be negatively associated with those of *Faecalibacterium* and overall *but*-containing taxa (both only RA) (Table 2). In the case of AXOS, the bifidogenic effect was negatively associated with the butyrate production potential and associated taxa.

## Discussion

Our pooled analyses demonstrated distinct target spectra for individual fibers that selectively promoted the growth of different members of gut microbiota. To perform pooled microbiome analyses, harmonization of data and associated analysis strategies is essential, which is exemplified by applying the same taxonomy for all studies under investigation. Bacterial classification can differ substantially between major databases, such as RDP^30^ and SILVA^31^. Furthermore, taxonomies are not constant, but are regularly adjusted based on new findings; the reclassification of *Eubacterium rectale* to *Agathobatcer rectale* as well as the separation of *Phocaeicola* taxa from *Bacteroides* are more recent examples^32^. In addition, bioinformatic procedures often vary between studies influencing results. While OTU based approaches were performed earlier by applying various sequence similarity cut-offs, more modern approaches yield amplicon sequence variants that provide a higher resolution^33^. Unfortunately, harmonization of studies is only possible to a certain degree, primarily encompassing bioinformatics and data analysis. Thus, whether the remaining discrepancies, such as the vast differences in abundances of propionate pathways in pre-intervention data observed, resulted from wet lab procedures applied or reflected true biological differences, such as individual subject characteristics, is unknown. For example age, that is known to affect microbial composition. We excluded studies solely focusing on children, however, no upper age limit was set due to a lack of data and any potential impact of age was, hence, not investigated here.

Based on our results, ITF and AXOS triggered a bifidogenic effect that has already been reported in the literature, also in the studies included here. *Bifidobacteria* have a high capacity to degrade polysaccharides^34^ and in vitro experiments verified their growth on ITF and AXOS ^35,36^. Hence, the bifidogenic effect is probably due to the direct utilization of the applied fibers. However, we showed that it was not a universal response to fibers (no increase upon RS treatment) and substantially differed in magnitude with higher stimulation in AXOS-treated individuals compared to results from interventions applying ITF. Those data suggest additional mechanisms are involved, such as nutrient competition with other members of gut microbiota, and stress the need to obtain quantitative insights in order to compare outcomes and comprehensively assess a fiber’s potential to stimulate certain taxa^34^.

Importantly, apart from substantiating the well-known bifidogenic effect, we discerned signals linked to key functions known to be beneficial for the host, namely, the production of the SCFA butyrate. Multiple studies have shown a butyrogenic effect of ITF and RS^37^ and our results suggest that this effect is due to the selective propagation of *but*-containing taxa. While both substrates triggered the proliferation of bacteria exhibiting this pathway, the individual taxa targeted were distinct, demonstrating the importance of taking functional redundancy into account when investigating host – microbe interactions^38^. A combination of two taxa, *Faecalibacterium* and *Anaerostipes*, primarily governed the response to ITF, whereas *Agathobacter* explained the butyrogenic effect of RS. Other butyrate producers, even those exhibiting *but*, were unaffected or declined. Selective stimulation of certain butyrate-producing taxa is more complex than that of *Bifidobacteria* and can occur at several levels. For instance, ITF is known to be degraded by *Faecalibacterium* strains rendering them direct targets for the prebiotic^39^. However, *Roseburia* decreased upon intervention despite its ability to grow on inulin in pure culture^35^ exemplifying that in vitro-based results are not always applicable to real-world settings. It should be noted that 16S rRNA gene-based results only allow investigations at the genus level and responses at the species/strain level, where degradation capabilities of specific substrates can greatly vary, were blind to our analyses. Additional stimulation of butyrate-producing taxa can be triggered by cross-feeding mechanisms. As mentioned in the introduction, it is known that *Bifidobacteria*-derived lactate can be consumed by certain taxa to produce butyrate and might be one explanation for *Anaerostipes* growth upon ITF treatment^15^, the responses of the two taxa on ITF were also positively correlated in the pooled analysis of this study. In the case of RS, where no bifidogenic effect was observed, the relative abundance of *Anaerostipes* declined and correlated with responses of *Bifidobacterium* as well. For AXOS, the abundance of *Anaerostipes* was unaffected despite the highest stimulation of *Bifidobacterium*. The model organism for the conversion of lactate to butyrate, *Anaerobutyricum* (formerly classified as *Eubacterium* (*hallii*))^40^, did not increase in its relative abundance in any study, and associations with responses of *Bifidobacterium* were incongruent between intervention types. The role of *Bifidobacterium*-derived lactate in stimulating a butyrogenic effect in treatments with ITF (and other substances), hence need further investigations. In general, lactate is a major fermentation end product of many bacteria and readily consumed by others, where its conversion to butyrate, and to propionate via the Pdiol pathway, are considered the major routes. Minor pathways such as the formation of propionate via the acrylate pathway^41^ or production of acetate during dissimilatory sulfate reduction exist as well^16^. More detailed analyses on a functional level focusing on lactate production as a whole and subsequent consumption need to be performed in order to provide quantitative answers on the fate of lactate in vivo upon interventions with prebiotics.

Another important metabolic cross-feeding mechanism in the context of this study is the need for extracellular acetate supply for the functioning of But and subsequent growth of taxa encoding this enzyme. It has been shown that major *but*-exhibiting taxa are net-acetate consumers, where acetate concentrations in media controlled the enzyme activity and growth rates of those bacteria^42^. Thus, the production of acetate by non-butyrate and non-propionate producers probably plays a major role in stimulating butyrate synthesis upon fiber treatment, as suggested earlier^7^. It is likely that increases in *Bifidobacterium* contribute to this effect^40^; however, it only represents one of many acetogenic members of gut microbiota, explaining why its responses did not directly correlate with the butyrate production potential. A third important mechanism for stimulating the growth of butyrogenic taxa is the availability of breakdown products derived from the extracellular degradation of fibers by so-called primary degraders. This is particularly important in the case of RS, where *R. bromii* and certain *Bifidobacteria* species, including *B. adolescentis* were demonstrated to act on those substrates, rendering them available to other taxa^7,38,43^. We speculate that the increase in *Agathobacter* was promoted by such a cross-feeding mechanism. The taxon can multiply on starch^35^ but cannot act on its resistant forms and, hence, most probably relies on the activity of primary degraders. The abundance of *Ruminococcus* did not significantly increase upon RS treatment, however, we found that higher concentrations of this taxon pre-intervention were key for *but*-exhibiting bacteria to increase in abundance. Interestingly, the opposite pattern was observed for ITF, where low initial *Ruminococcus* relative abundances were associated with a positive response of *but*-containing bacteria. These results suggest that *Ruminococcus*, in contrast to studies that applied RS, did not act as a cross-feeder in the case of ITF. The initial *Prevotella* concentrations were negatively correlated with an increase in the butyrogenic potential for both ITF and RS. The exact mechanism is unknown, however, it is likely that *Prevotella* acts as a direct nutrient competitor given its high ability to degrade diverse polysaccharides^44^. Nutrient competition might also explain the significant (p < 0.05, p < 0.1 (*lmer*)) negative association between responses of this taxon with *but*-containing bacteria on both ITF and RS (data not shown). In summary, our data indicate that *Ruminococcus* and *Prevotella* predispose individuals to respond to ITF and RS in terms of the butyrate production potential. The bifidogenic effect was barely correlated and largely represented a separate response.

The abundance of the entire AcCoA pathway was hardly affected by any intervention, and the route via *buk* even declined in several cases. However, *but*-carrying taxa are the most potent butyrate producers, especially when compared with bacteria that exhibit the AcCoA pathway lacking both *but* and *buk* as terminal enzymes. The latter taxa are often linked to protein degradation yielding less butyrate^45,46^. Stratifications based on the terminal enzyme are important to properly assess the butyrate production potential. Detailed in vitro studies will further specify the contributions of each of the three biochemically distinct groups to overall butyrate production in quantitative terms.

For propionate, no convincing signals were detected in the pooled analyses. The Suc pathway was hardly affected by the interventions, along with its main members; the Pdiol pathway strongly declined with all fiber treatments. Production of butyrate is tightly linked to the pathway presence in bacteria, especially for those carrying *but*, whereas propionate synthesis is more prone to metabolic flexibility, particularly for the Pdiol pathway, which does not represent an essential biochemical route for the respective bacteria^12^. Thus, our analyses should not be interpreted as the inability of fibers to act on propionate production, but should encourage investigations on multi(omics) levels to discern mechanisms in detail, taking bacterial physiology into account^26,47^.

We and others have demonstrated that data on relative abundances of SCFA pathways and respective taxa correlated with SCFA composition in vitro and in vivo^12,48^. Furthermore, a recent meta-analysis performed by LaBouyer and colleagues showed that the proportion of butyrate increases as total fecal SCFA concentrations increase due to elevated overall fermentation of fibers^49^. The authors explained this phenomenon by a decrease in pH that stimulates production of butyrate through acetate consumption of *but*-containing taxa and is in line with results presented in Kircher and colleagues^12^. Unfortunately, information on SCFA proportions during interventions were only available from two studies (Supplementary Figure 2). In this context it should also be noted that fecal SCFA concentrations might only partly reflect overall production as the majority of SCFAs ( > 95%) is readily consumed by the host and other bacteria and residual levels in feces is confounded by other factors such as transit time. On this line, it was demonstrated that fecal and plasma concentrations of SCFA do not correlate^50^. Thus, obtaining comprehensive insights into SCFA production in vivo and how this is governed by gut microbiota functioning in quantitative terms remains a challenging task.

In conclusion, our results demonstrated that ITF and RS selectively stimulated bacterial taxa, increasing the butyrate production potential, whereas a strong bifidogenic effect was observed for AXOS. Furthermore, our results provide crucial information on the common understanding that stratification based on microbiota composition is key to providing effective, personalized treatment. These findings will stimulate intervention designs by applying hypothesis-driven stratification of individuals. Coupling in vivo insights with comprehensive in vitro testing and advanced metagenomic procedures that allow the linkage of functions at the genome level will further progress the field.

## Methods

### Literature searching strategy

A review by Swanson and colleagues (2020)^2^ served as a guideline for searching eligible studies; additional string searches were performed on the PubMed Central database in Spring 2022, systematically searching dietary intervention studies, where microbiota composition was monitored. All studies until (including) 2021 were considered. The inclusion criteria were as follows: (1) human intervention study applying a single defined fiber; (2) (randomized) human trials including a placebo/control group; (3) adults; and (4) 16S rRNA gene sequencing data derived from an Illumina platform. Raw sequencing data and associations with interventions were retrieved from the European Nucleotide Archive (ENA). If the respective data were not available, queries to the corresponding authors were sent and, if required, queries were resent after a few weeks. Finally, only studies that encompassed at least two individual studies of a fiber type were included.

### Bioinformatics

The obtained data were treated with DADA2 (v1.20), yielding amplicon sequence variants (ASVs) that were classified based on RDP^30,33^. ASVs were agglomerated at the genus level, which represents the lowest taxonomic resolution possible to compare studies targeting distinct variable regions of the 16S rRNA gene. Furthermore, predictions of SCFA-forming pathways, including terminal genes *but* and *buk* were performed as described previously^12^. All data are expressed as relative abundance data. To focus our analyses on the most relevant (abundant) aspects, analyses of individual taxa were only conducted for major members associated with each pathway based on^12^ as well as the most abundant non-butyrate, non-propionate-synthesizing taxa.

### Data analysis

Before comparing outcomes of studies, data were harmonized to be represented by two time points (pre- and post-intervention for each subject) for the intervention and placebo/control groups. Cross-over designs were not considered; average values were calculated for studies that obtained several samples during the intervention period. For Maier et al. (2017)^26^ we regarded the low RS dose as the control group. For Healey et al. (2018)^18^ and Reimer et al. (2020)^24^ only the groups receiving high and moderate doses, respectively, were considered. Subjects receiving probiotics were omitted from the study of Ford et al. (2019)^21^.

Subsequently, changes post-intervention relative to the pre-intervention data were calculated, referred to as relative response (RR) data that were capped at +/- 100% in order to lower variances. The absolute change post- versus pre-intervention based on relative abundance data, defined as the relative abundance change (RA), was also calculated. Outcomes of the intervention group compared to the respective placebo/control group were then analyzed in R (v4.2.2) using linear mixed-effect models (function *lmer* of the *lme4* package (v1.1–27.1)) including studies as a random effect (random intercept and random slope). The results of the two groups of individual studies were compared using the *lm* function. Results are expressed as the estimated effect size (regression coefficient) along with 95% confidence intervals that were retrieved via the *confint* function. Data were visualized via the package *ggplot2* (v3.3.5). Stratifications of *Ruminococcus* relative abundance pre-intervention (based on the median and quartiles) were performed for each study individually. Similar for *Prevotella*, where data were stratified into two groups based on their relative abundance (cut-off of 1%) pre-intervention.

The institutional review board of Hannover Medical School approved the waiver of informed consent as this study is based on secondary analyses of already published data without any patient reference.

### Supplementary information

Supplementary Information
