# Supplementary material for: Investigating the response of the butyrate production potential to major fibers in dietary intervention studies
Source: NPJ Biofilms Microbiomes. 2024 Jul 30;10:63. doi: 10.1038/s41522-024-00533-5 (PMC11289085; doi:10.1038/s41522-024-00533-5)
Supplement: Supplementary file 1 — Supplementary Information [file 41522_2024_533_MOESM1_ESM.pdf]

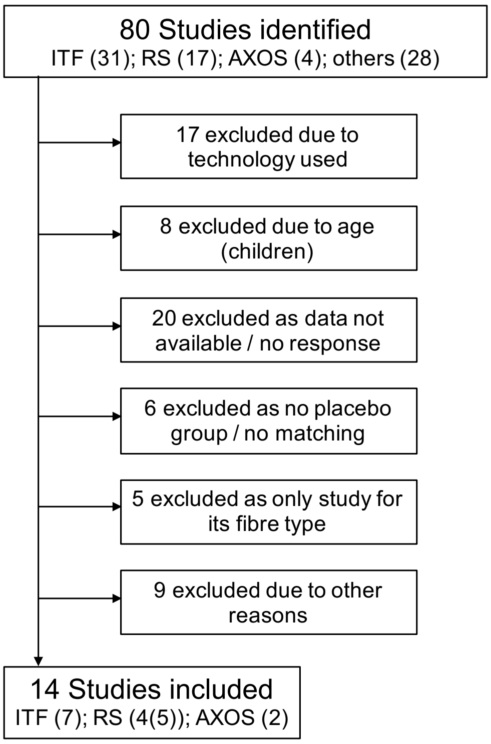

Supplementary Figure 1. Flow chart displaying the screening process to identify eligible studies.

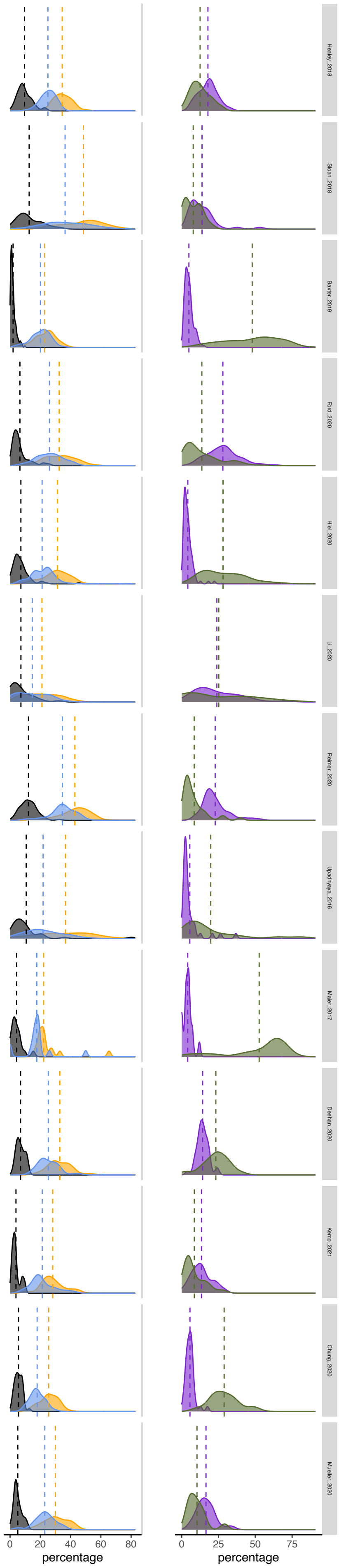

Supplementary Figure 2. Relative abundances of predicted pathways pre-intervention for all studies are given.

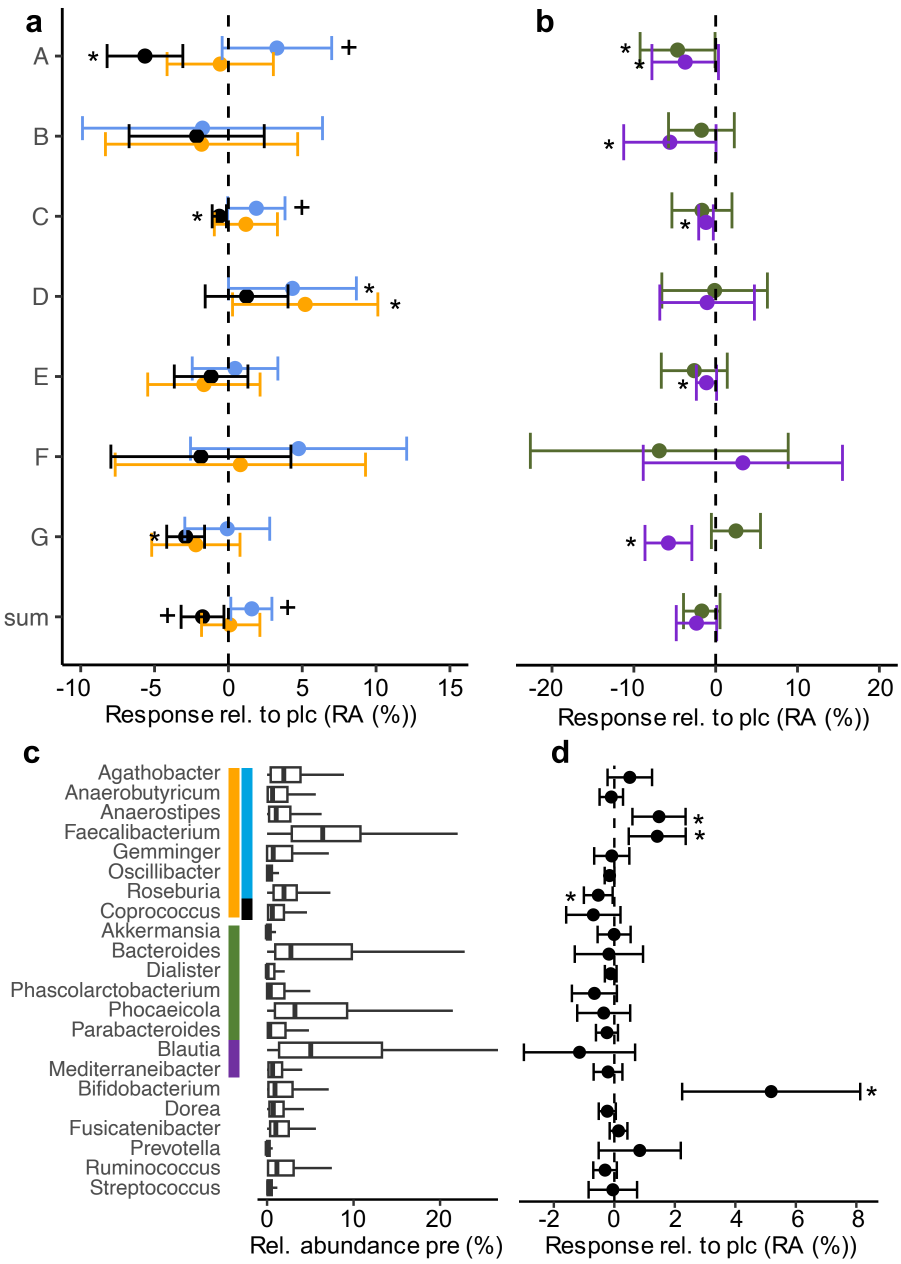

Supplementary Figure 3. Intervention outcomes based on Inulin-type fructans (ITF). Panel a shows the response (compared with placebo) based on relative abundance changes (RA) of the main butyrate pathway (orange) along with the two terminal enzymes *but* (light blue) and *buk* (black). Responses of the two propionate-forming pathways (Suc: green and Pdiol: violet) are given in panel b. sum: results based on pooled analyses. For detailed characteristics of individual studies see Table 1. Below (panel c) relative abundances pre-intervention and responses relative to pre-intervention (compared with placebo) (d) of major taxa associated with individual pathways are given. The estimated effect sizes from linear mixed effect models including studies as a random effect (*lmer*) along with their 95 % confidence intervals are given. *, +: p<0.05, p<0.1 compared with placebo.

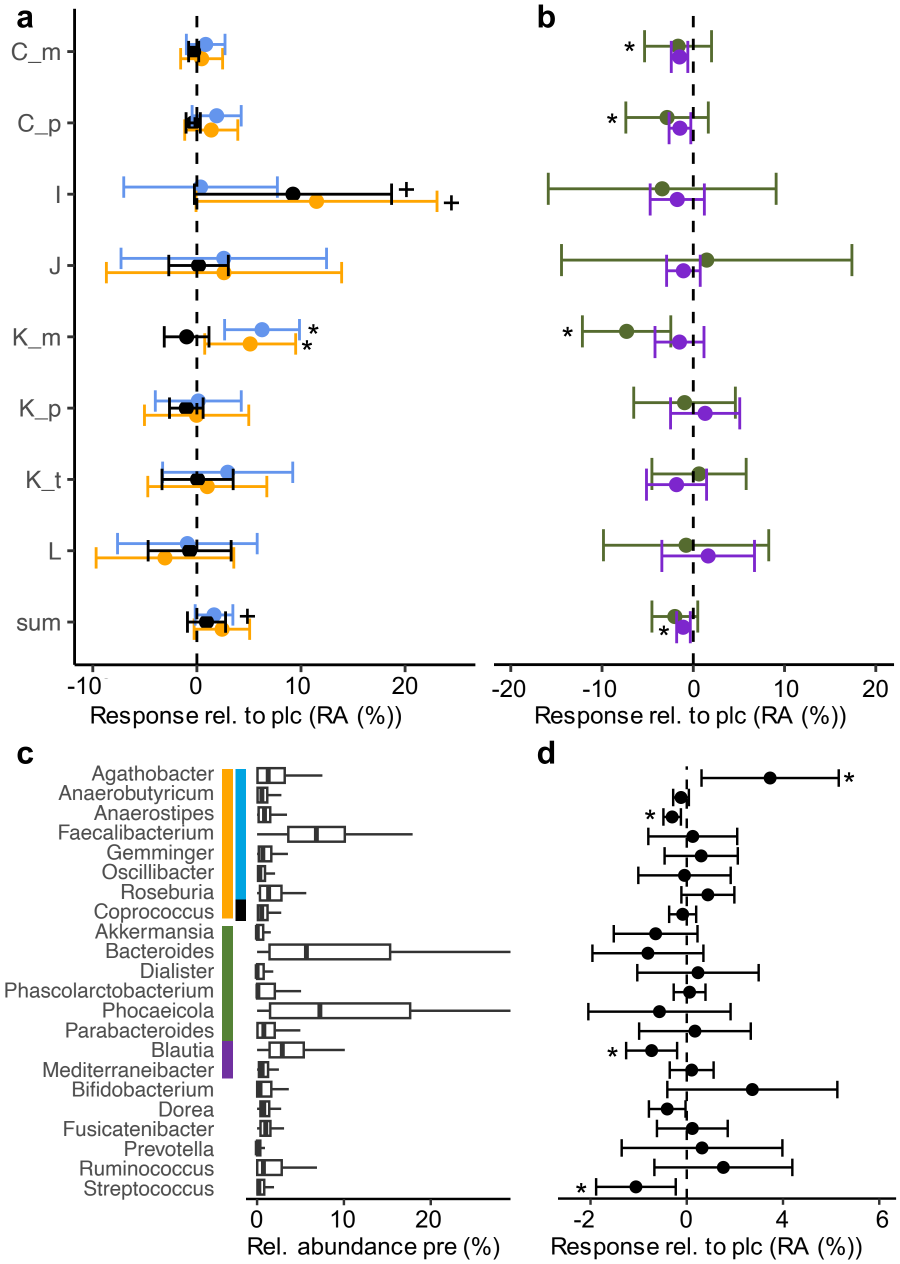

Supplementary Figure 4. Intervention outcomes based on Resistant Starches (RS). Panel a shows the response (compared with placebo) based on relative abundance changes (RA) of the main butyrate pathway (orange) along with the two terminal enzymes *but* (light blue) and *buk* (black). Responses of the two propionate-forming pathways (Suc: green and Pdiol: violet) are given in panel b. sum: results based on pooled analyses. For study C and K groups treated with starches from different sources (m: maize; p: potato; t; tapioca) were included as separate studies; for detailed characteristics of individual studies see Table 1. Below (panel c) relative abundances pre-intervention and responses relative to pre-intervention (compared with placebo) (d) of major taxa associated with individual pathways are given. The estimated effect sizes from linear mixed effect models including studies as a random effect (*lmer*) along with their 95 % confidence intervals are given. *, +: $p<0.05$, $p<0.1$ compared with placebo.

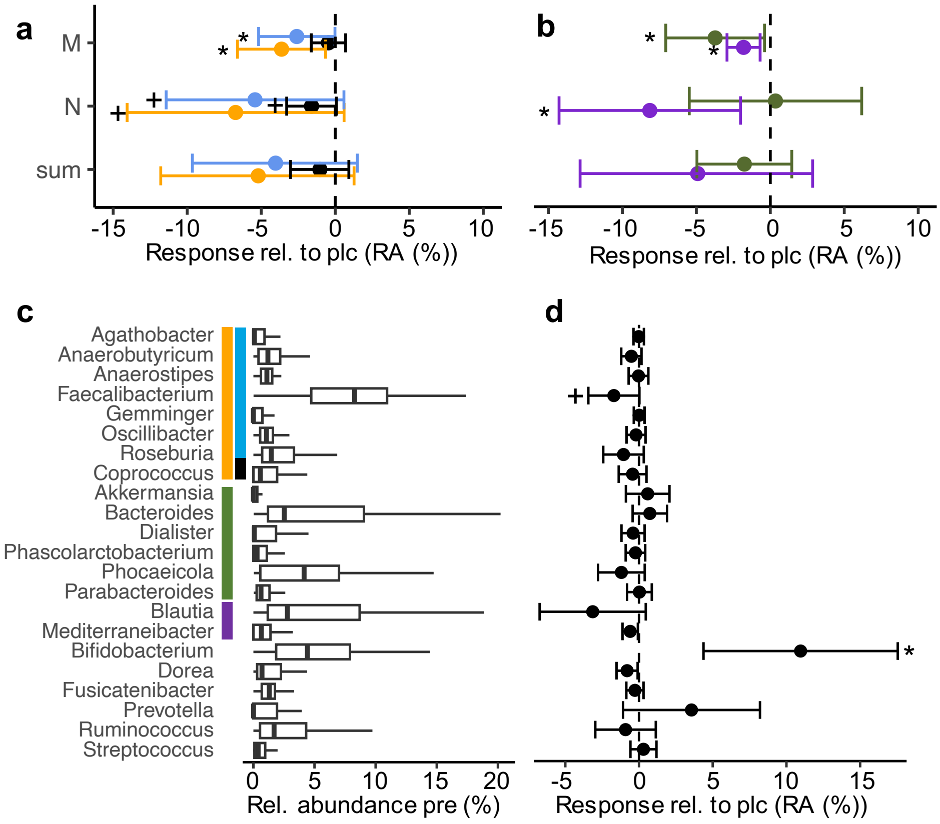

Supplementary Figure 5. Intervention outcomes based on Arabinoxylan-Oligosaccharides (AXOS). Panel a shows the response (compared with placebo) based on relative abundance changes (RA) of the main butyrate pathway (orange) along with the two terminal enzymes *but* (light blue) and *buk* (black). Responses of the two propionate-forming pathways (Suc: green and Pdiol: violet) are given in panel b. sum: results based on pooled analyses. For detailed characteristics of individual studies see Table 1. Below (panel c) relative abundances pre-intervention and responses relative to pre-intervention (compared with placebo) (d) of major taxa associated with individual pathways are given. The estimated effect sizes from linear mixed effect models including studies as a random effect (*lmer*) along with their 95 % confidence intervals are given. *, +: p<0.05, p<0.1 compared with placebo.

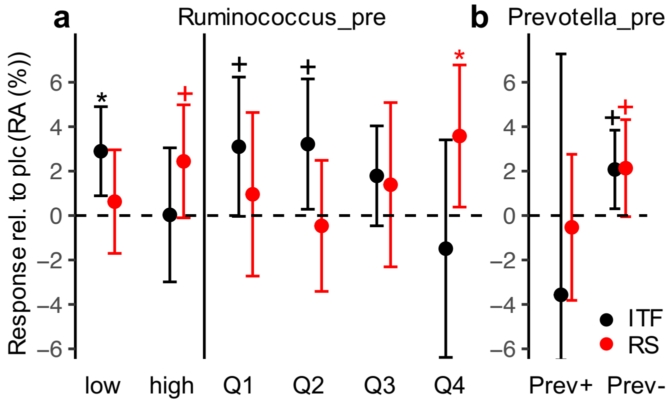

Supplementary Figure 6. Relative abundances of *Ruminococcus* and *Prevotella* pre-intervention affecting increase of *but*-containing bacteria upon ITF and RS treatment. Panel a give results after stratification by *Ruminococcus* abundance based on median abundances (low: below median, high: above median) and quartile abundances, whereas panel b shows outcomes after stratification for *Prevotella* abundance, where Prev+ refers to samples comprising >= 1 % *Prevotella* relative abundance pre-intervention (Prev- represent all other samples). Results display relative abundance changes (RA) of *but*-exhibiting bacteria after intervention compared with placebo. Stratifications were performed for each study individually. The estimated effect sizes from linear mixed effect models including studies as a random effect (*lmer*) along with their 95 % confidence intervals are given. *, +: p<0.05, p<0.1 compared with placebo.

Supplementary Table 1. Response of bacteria exhibiting SCFA pathways to ITF treatment compared with controls. The estimate along with the 95 % confidence interval is given. Results based on both responses relative to pre-intervention and relative abundance changes are given for all studies and the pooled analysis.

		relative response				***relative abundance change***			
**pathway**	**dataset**	**estimate**	**0,025**	**0,975**	**p**	**estimate**	**0,025**	**0,975**	**p**
AcCoA	A	-2,678	-16,498	11,142	0,697	-0,554	-4,153	3,045	0,757
AcCoA	B	-4,356	-22,947	14,235	0,637	-1,818	-8,327	4,692	0,574
AcCoA	C	3,796	-10,403	17,995	0,597	1,184	-0,954	3,322	0,274
AcCoA	D	19,721	-0,448	39,890	**0,055**	5,200	0,279	10,121	**0,039**
AcCoA	E	-5,117	-21,573	11,339	0,538	-1,660	-5,465	2,145	0,389
AcCoA	F	5,925	-45,468	57,317	0,815	0,810	-7,666	9,286	0,846
AcCoA	G	-6,555	-15,567	2,457	0,151	-2,204	-5,193	0,786	0,146
But	A	15,066	-3,151	33,283	0,102	3,285	-0,423	6,994	0,081
But	B	-10,803	-34,393	12,788	0,359	-1,754	-9,881	6,373	0,664
But	C	8,688	-5,373	22,750	0,223	1,890	-0,052	3,833	0,056
But	D	20,328	-3,850	44,506	**0,097**	4,337	0,009	8,666	**0,050**
But	E	7,510	-11,401	26,421	0,432	0,451	-2,449	3,350	0,758
But	F	35,793	-15,287	86,873	0,162	4,753	-2,566	12,072	0,194
But	G	0,974	-10,395	12,343	0,865	-0,078	-2,953	2,797	0,957
Buk	A	-66,031	-96,722	-35,339	**0,000**	-5,650	-8,215	-3,085	**0,000**
Buk	B	-17,595	-57,148	21,958	0,373	-2,149	-6,725	2,427	0,347
Buk	C	-45,684	-72,899	-18,469	**0,001**	-0,615	-1,097	-0,133	**0,013**
Buk	D	12,494	-24,951	49,940	0,505	1,232	-1,573	4,036	0,381
Buk	E	-14,896	-42,285	12,492	0,283	-1,171	-3,663	1,321	0,353
Buk	F	-37,823	-97,788	22,143	0,207	-1,863	-7,959	4,234	0,536
Buk	G	-31,970	-47,460	-16,481	**0,000**	-2,896	-4,178	-1,614	**0,000**
Suc	A	-33,323	-77,831	11,186	0,138	-4,656	-9,235	-0,077	**0,046**
Suc	B	-16,452	-63,618	30,714	0,484	-1,742	-5,761	2,278	0,385
Suc	C	-3,668	-15,962	8,627	0,555	-1,679	-5,349	1,992	0,366
Suc	D	1,807	-40,018	43,632	0,931	-0,129	-6,579	6,321	0,968
Suc	E	-10,368	-29,097	8,361	0,274	-2,615	-6,646	1,417	0,201
Suc	F	-21,921	-85,763	41,921	0,488	-6,892	-22,648	8,864	0,378
Suc	G	3,295	-31,753	38,343	0,852	2,476	-0,526	5,478	0,105
Pdiol	A	-32,363	-59,354	-5,372	**0,020**	-3,723	-7,789	0,343	**0,072**
Pdiol	B	-43,218	-76,800	-9,635	**0,013**	-5,597	-11,239	0,045	**0,052**
Pdiol	C	-32,992	-56,304	-9,681	**0,006**	-1,181	-2,074	-0,287	**0,010**
Pdiol	D	-8,069	-34,876	18,738	0,547	-1,049	-6,831	4,732	0,716
Pdiol	E	-24,693	-49,183	-0,203	**0,048**	-1,124	-2,359	0,112	**0,074**
Pdiol	F	21,940	-32,391	76,271	0,415	3,322	-8,853	15,497	0,581
Pdiol	G	-32,248	-47,828	-16,668	**0,000**	-5,775	-8,637	-2,913	**0,000**
AcCoA	sum	0,590	−6,277	7,458	0,866	0,098	−1,738	1,933	0,920
But	sum	9,159	1,560	16,758	**0,019**	1,583	0,144	3,022	**0,080**
Buk	sum	-28,201	−45,840	−10,562	**0,023**	-1,753	−3,203	−0,303	**0,065**
Suc	sum	-8,915	−20,590	2,760	0,137	-1,656	−3,614	0,303	0,207
Pdiol	sum	-24,906	−37,654	−12,158	**0,011**	-2,348	−4,816	0,120	0,108

Supplementary Table 2. SCFA concentrations measured in individual studies along with compositional changes originally detected.

Akrn	SCFA	Composition change originally reported
A	No sig. effect<br>(Acet, Butyr, Prop ↑)	Bifidobacterium ↑ Coprococcus ↓, Dorea ↓, Ruminococcus ↓, Oscillospira ↓, Faecalibacterium ↑ (trend)
B	No sig. effect<br>(Acet, Butyr, Prop ↓)	Bifidobacterium ↑, Butyricimonas↑, Paraprevotella ↑, Lactobacillus ↑, Catenibacterium ↑, Parabacteroides ↓, IncertaeSedis ↓, Ruminococcus ↓, Turicibacter ↓, Anaerostipes ↓, Blautia ↓
C	No sig. effect<br>(Acet ↑ (p<0.1), Butyr, Prop ↑)	Bifidobacterium ↑, Anaerostipes ↑
D	Not measured	none
E	Not measured	Bifidobacterium ↑, Catenibacterium ↑, Desulfovibrio ↓, Roseburia ↓, Dorea ↑, Erysipelptrichaceae incertae sedis↑ , Escherichia/Shigella ↑, Lactobacillus↑ , Butyricimonas ↓, Clostridium sensu stricto ↓, Clostridium cluster XIVa ↓
F	Not measured	Bacteroides thetaiotaomicron ↓
G	No sig. effect<br>(Acet, Butyr, Prop ↑)	Bifidobacterium ↑, Cellulomonas↑, Nesterenkonia ↑, Brevibacterium ↑, Lachnospira ↓, Oscillospira ↓, Ruminococcus ↓
C_m, C_p	C_p: Acet and Butyr ↑<br>(Prop ↓)<br>(C_m: Butyr ↑, Acet ↓)	C_p: Bifidobacterium faecale/adolescentis/stercois ↑<br>C_m: Ruminococcus bromii ↑
I	Butyr and Prop ↑<br>(Acet ↑)	Ruminococcus ↑, Blautia ↑, Bacteroides ↑, Oscillospira ↑, Parabacteroides ↑, Blautia glucerasea ↑, Christensenella minuta↑, Eubacterium oxidoreducens ↑, Oscillospira sp. ↑, Ruminococcus lactaris ↑, Parabacteroides distasonis↑ , Enterococcus casseliflavus ↓, Streptococcus cristatus↓
J	No sig. effect<br>(Butyr, Prop ↑)	Faecalibacterium ↑ , Roseburia ↑, Ruminococcus ↑, F. Prausnitzii ↑, Preveotellaceae ↑, Eubacterium rectale ↑, Roseburia faecis ↑, Akkermansia muciniphila ↑
K_m, K_p, K_t	K_m: Butyr ↑ (Prop ↓)<br>K_t: Prop ↑ (Acet, Butyr ↑)<br>(K_p: Acet, Prop ↓)	K_m: OTUs: Eubacterium rectale ↑, Oscillibacter spp. ↑, Ruminococcus spp. ↑, Anaeromassilibacillus spp. ↑, Ruminococcus callidus↓ , Agathobaculum butyriciproducens ↓, Adlercreutzia equolifaciens↓<br>K_p: none<br>K_t: Genus: Parabacteroides ↑, Ruminococcaceae (unclassified genus)↓ OTUs:Parabacteroides distasonis ↑, Parabacteroides spp. ↑, Faecalibacterium prausnitzii ↑, Eisenbergiella spp. ↑, Eubacterium hallii ↓, Clostridium viride↓
L	Not measured	Oscillosperaceae ↑, Roseburia ↑, Ruminococcus gauvreauii ↑, Ruminococcus champanellens ↓, Dialister, Coprococcus ↓
M	No sig. effect<br>(Acet, Butyr, Prop ↑)	Bifidobacterium ↑
N	No sig. effect<br>(Acet, Prop ↑ (only *Prevotella*+))	Bifidobacterium ↑, Akkermansia ↑, Prevotellaceae NK3831 group ↑, Lactobacillus ↑, Blautia ↓, Eubacterium hallii group ↓, Coriabacteriaceau UCG-003 ↓, Dorea ↓

Supplementary Table 3. Response of individual taxa to ITF treatment compared with controls. The estimate along with the 95 % confidence interval is given. Results based on both responses relative to pre-intervention and relative abundance changes are given for all studies and the pooled analysis.

		relative response				*relative abundance change*			
**taxon**	**dataset**	**estimate**	**0,025**	**0,975**	**p**	**estimate**	**0,025**	**0,975**	**p**
Agathobacter	A	2,824	-41,534	47,183	0,898	0,264	-1,602	2,130	0,776
Agathobacter	B	6,899	-48,966	62,764	0,804	0,913	-5,495	7,321	0,774
Agathobacter	C	21,668	-6,477	49,814	0,130	0,946	0,296	1,596	**0,005**
Agathobacter	D	56,906	11,828	101,983	**0,015**	0,727	-0,989	2,443	0,398
Agathobacter	E	2,585	-29,591	34,760	0,874	-0,110	-1,158	0,938	0,836
Agathobacter	F	-21,185	-62,225	19,855	0,299	1,556	-1,356	4,468	0,283
Agathobacter	G	8,079	-23,358	39,516	0,610	0,373	-0,965	1,711	0,580
Akkermansia	A	-10,072	-58,087	37,942	0,673	0,036	-0,192	0,265	0,749
Akkermansia	B	-10,493	-56,205	35,219	0,644	0,067	-0,419	0,553	0,781
Akkermansia	C	10,560	-17,028	38,148	0,449	-0,032	-0,614	0,551	0,914
Akkermansia	D	-31,312	-77,571	14,948	0,179	0,945	-2,604	4,494	0,594
Akkermansia	E	10,416	-18,660	39,492	0,479	-0,522	-1,376	0,332	0,228
Akkermansia	F	-24,645	-63,460	14,171	0,204	-0,008	-0,044	0,028	0,650
Akkermansia	G	-18,972	-58,287	20,342	0,339	-0,200	-1,256	0,856	0,707
Anaerobutyricum	A	-7,027	-40,942	26,887	0,677	-0,134	-0,739	0,470	0,655
Anaerobutyricum	B	-36,625	-82,710	9,460	0,116	-0,171	-0,570	0,229	0,392
Anaerobutyricum	C	-22,064	-49,517	5,389	0,114	-0,076	-0,231	0,078	0,329
Anaerobutyricum	D	-14,155	-49,996	21,687	0,430	-0,221	-2,140	1,699	0,818
Anaerobutyricum	E	14,964	-18,072	48,000	0,371	0,026	-0,023	0,075	0,292
Anaerobutyricum	F	-5,379	-57,978	47,220	0,836	1,001	-1,990	3,993	0,499
Anaerobutyricum	G	-24,199	-45,977	-2,421	**0,030**	-0,811	-1,539	-0,083	**0,030**
Anaerostipes	A	75,282	36,820	113,743	**0,000**	3,872	2,087	5,657	**0,000**
Anaerostipes	B	23,294	-30,245	76,833	0,383	0,061	-0,780	0,902	0,883
Anaerostipes	C	41,385	13,633	69,138	**0,004**	1,034	0,524	1,544	**0,000**
Anaerostipes	D	33,749	-7,936	75,433	0,110	2,067	-0,593	4,727	0,125
Anaerostipes	E	64,479	35,885	93,072	**0,000**	0,561	0,335	0,786	**0,000**
Anaerostipes	F	-2,017	-68,437	64,403	0,951	1,367	0,023	2,710	**0,046**
Anaerostipes	G	45,620	19,703	71,537	**0,001**	1,885	0,659	3,111	**0,003**
Bacteroides	A	-32,210	-84,155	19,735	0,217	-1,382	-3,098	0,335	0,111
Bacteroides	B	-25,219	-76,500	26,061	0,325	-0,864	-2,327	0,600	0,239
Bacteroides	C	5,318	-11,930	22,566	0,542	0,332	-1,547	2,211	0,726
Bacteroides	D	3,765	-40,979	48,509	0,866	0,492	-2,169	3,152	0,711
Bacteroides	E	-4,113	-34,144	25,919	0,786	-0,414	-2,211	1,382	0,648
Bacteroides	F	-27,565	-90,772	35,642	0,379	-3,542	-12,986	5,902	0,449
Bacteroides	G	17,697	-20,968	56,362	0,365	1,622	0,240	3,004	**0,022**
Bifidobacterium	A	82,984	37,900	128,067	**0,001**	8,400	4,766	12,034	**0,000**
Bifidobacterium	B	112,515	68,331	156,698	**0,000**	12,579	7,217	17,942	**0,000**
Bifidobacterium	C	53,869	27,402	80,336	**0,000**	2,693	0,767	4,620	**0,007**
Bifidobacterium	D	19,298	-26,543	65,140	0,401	1,212	-0,628	3,053	0,191
Bifidobacterium	E	75,091	45,132	105,050	**0,000**	3,287	1,414	5,159	**0,001**
Bifidobacterium	F	4,614	-58,843	68,072	0,883	2,326	-4,476	9,127	0,489
Bifidobacterium	G	64,817	31,858	97,777	**0,000**	7,673	5,332	10,014	**0,000**
Blautia	A	-18,190	-49,939	13,559	0,253	-1,008	-4,451	2,436	0,557
Blautia	B	-50,902	-88,777	-13,027	**0,010**	-4,415	-9,939	1,109	0,114
Blautia	C	-23,120	-47,657	1,418	**0,064**	-0,736	-1,403	-0,070	**0,031**
Blautia	D	-8,264	-39,745	23,216	0,599	-1,129	-6,314	4,056	0,663
Blautia	E	4,226	-23,437	31,888	0,762	0,295	-0,203	0,793	0,242
Blautia	F	-3,226	-67,562	61,109	0,919	3,288	-5,629	12,206	0,456
Blautia	G	-22,968	-41,223	-4,714	**0,014**	-3,104	-5,748	-0,459	**0,022**
Coprococcus	A	-56,265	-95,252	-17,279	**0,006**	-2,409	-3,990	-0,828	**0,004**
Coprococcus	B	-59,488	-105,328	-13,649	**0,012**	-2,580	-4,690	-0,470	**0,018**
Coprococcus	C	-41,433	-67,038	-15,829	**0,002**	-0,271	-0,494	-0,047	**0,018**
Coprococcus	D	-5,828	-42,807	31,152	0,752	0,362	-0,203	0,927	0,203
Coprococcus	E	-7,662	-37,981	22,658	0,617	0,192	-0,240	0,625	0,380
Coprococcus	F	-2,805	-37,360	31,749	0,869	-0,057	-0,398	0,283	0,734
Coprococcus	G	-32,634	-56,684	-8,583	**0,009**	-0,484	-1,182	0,214	0,171

Dialister	A	-16,808	-55,843	22,228	0,389	-0,558	-1,129	0,013	**0,055**
Dialister	B	12,242	-29,408	53,892	0,555	-0,068	-1,459	1,322	0,921
Dialister	C	-0,299	-27,615	27,018	0,983	-0,013	-0,230	0,204	0,906
Dialister	D	-14,404	-40,150	11,342	0,266	-0,397	-1,029	0,235	0,212
Dialister	E	12,176	-12,253	36,605	0,325	0,098	-0,049	0,246	0,189
Dialister	F	4,261	-48,633	57,155	0,870	0,299	-0,107	0,705	0,143
Dialister	G	-30,923	-58,843	-3,004	**0,030**	-0,297	-0,600	0,007	**0,055**
Dorea	A	2,139	-37,201	41,479	0,913	0,248	-0,527	1,024	0,520
Dorea	B	5,598	-37,592	48,788	0,794	0,035	-0,459	0,529	0,887
Dorea	C	-21,103	-47,724	5,518	0,119	-0,106	-0,293	0,080	0,259
Dorea	D	5,672	-28,318	39,663	0,738	-0,103	-0,743	0,536	0,746
Dorea	E	2,288	-25,998	30,574	0,873	-0,136	-0,500	0,228	0,460
Dorea	F	10,985	-48,507	70,478	0,708	-1,492	-4,640	1,656	0,340
Dorea	G	-25,380	-43,774	-6,985	**0,008**	-0,373	-0,815	0,069	**0,097**
Faecalibacterium	A	29,289	-1,509	60,088	**0,062**	2,526	0,229	4,822	**0,032**
Faecalibacterium	B	5,655	-42,781	54,091	0,814	2,400	-3,634	8,435	0,425
Faecalibacterium	C	10,449	-9,650	30,548	0,304	1,136	-0,104	2,376	**0,072**
Faecalibacterium	D	44,713	6,972	82,454	**0,021**	3,098	0,862	5,334	**0,008**
Faecalibacterium	E	17,263	-11,873	46,399	0,242	0,505	-1,064	2,074	0,524
Faecalibacterium	F	-0,244	-63,745	63,256	0,994	0,124	-5,314	5,562	0,963
Faecalibacterium	G	10,726	-17,607	39,059	0,453	1,183	-1,115	3,480	0,308
Fusicatenibacter	A	-4,088	-48,884	40,709	0,854	-0,313	-1,748	1,123	0,661
Fusicatenibacter	B	-11,773	-59,098	35,551	0,617	0,313	-1,172	1,799	0,671
Fusicatenibacter	C	8,821	-19,857	37,500	0,543	0,005	-0,235	0,245	0,966
Fusicatenibacter	D	31,770	-4,020	67,560	**0,080**	0,574	-0,158	1,306	0,121
Fusicatenibacter	E	23,321	-2,243	48,886	**0,073**	0,179	-0,077	0,435	0,169
Fusicatenibacter	F	-16,738	-69,282	35,806	0,519	0,375	-0,890	1,640	0,548
Fusicatenibacter	G	1,241	-20,845	23,326	0,911	0,092	-0,661	0,846	0,808
Gemminger	A	-28,047	-66,059	9,964	0,143	-1,019	-2,262	0,223	0,105
Gemminger	B	40,048	-5,973	86,069	**0,086**	1,450	-2,671	5,572	0,480
Gemminger	C	-1,949	-27,386	23,487	0,879	-0,152	-0,346	0,042	0,123
Gemminger	D	-4,251	-38,150	29,648	0,802	0,418	-1,816	2,652	0,708
Gemminger	E	6,057	-23,380	35,494	0,684	0,057	-0,306	0,420	0,756
Gemminger	F	-46,667	-84,243	-9,090	**0,017**	-0,627	-2,850	1,596	0,568
Gemminger	G	-16,439	-44,793	11,916	0,252	-0,781	-1,633	0,070	**0,072**
Mediterraneibacter	A	-30,054	-74,353	14,245	0,178	-1,168	-2,349	0,013	**0,052**
Mediterraneibacter	B	-30,595	-77,355	16,165	0,193	-0,273	-1,132	0,585	0,522
Mediterraneibacter	C	-37,410	-64,490	-10,331	**0,007**	-0,131	-0,327	0,066	0,189
Mediterraneibacter	D	-2,830	-40,822	35,162	0,881	-0,035	-1,004	0,935	0,943
Mediterraneibacter	E	-7,910	-40,648	24,827	0,632	-0,007	-0,096	0,081	0,869
Mediterraneibacter	F	12,184	-52,499	76,868	0,703	1,242	-0,343	2,827	0,120
Mediterraneibacter	G	-70,508	-96,489	-44,528	**0,000**	-0,906	-1,363	-0,449	**0,000**
Oscillibacter	A	-34,543	-82,723	13,636	0,155	-0,431	-0,999	0,137	0,133
Oscillibacter	B	-41,436	-87,723	4,852	**0,078**	-0,469	-1,340	0,402	0,281
Oscillibacter	C	-12,251	-42,174	17,671	0,418	-0,143	-0,311	0,025	**0,094**
Oscillibacter	D	-44,757	-82,842	-6,671	**0,022**	-0,176	-0,405	0,052	0,127
Oscillibacter	E	14,388	-14,207	42,983	0,320	0,080	-0,066	0,226	0,281
Oscillibacter	F								
Oscillibacter	G	2,429	-22,888	27,746	0,849	-0,008	-0,087	0,072	0,849
Parabacteroides	A	-31,170	-85,341	23,002	0,251	-0,215	-0,504	0,074	0,140
Parabacteroides	B	-19,709	-54,413	14,995	0,257	-0,170	-0,344	0,004	**0,055**
Parabacteroides	C	-28,794	-55,222	-2,366	**0,033**	-0,417	-0,833	0,000	**0,050**
Parabacteroides	D	-22,620	-69,432	24,192	0,335	0,093	-0,266	0,452	0,603
Parabacteroides	E	-14,134	-44,123	15,854	0,352	-0,525	-2,008	0,958	0,483
Parabacteroides	F	9,188	-53,575	71,951	0,767	-0,107	-1,522	1,308	0,878
Parabacteroides	G	-21,244	-58,684	16,196	0,262	0,016	-0,100	0,133	0,779
Phascolarctobacterium	A	-37,577	-76,198	1,044	**0,056**	-0,332	-0,944	0,280	0,279
Phascolarctobacterium	B	-21,567	-68,194	25,061	0,354	-1,094	-2,465	0,276	0,114
Phascolarctobacterium	C	4,014	-20,272	28,299	0,743	-0,332	-0,701	0,037	**0,077**
Phascolarctobacterium	D	-13,909	-51,649	23,832	0,462	-0,028	-1,215	1,160	0,962
Phascolarctobacterium	E	1,132	-23,848	26,113	0,928	-0,390	-1,223	0,443	0,355
Phascolarctobacterium	F	-17,965	-72,319	36,388	0,504	-3,091	-6,517	0,334	**0,075**
Phascolarctobacterium	G	5,103	-23,155	33,360	0,720	0,117	-0,190	0,425	0,450

Phocaeicola	A	-15,983	-66,765	34,799	0,528	-0,376	-2,426	1,673	0,712
Phocaeicola	B	-19,593	-71,629	32,443	0,450	-0,233	-1,798	1,332	0,764
Phocaeicola	C	-4,370	-21,826	13,087	0,620	-0,567	-2,880	1,746	0,627
Phocaeicola	D	16,451	-32,351	65,252	0,500	-0,705	-3,726	2,315	0,640
Phocaeicola	E	-0,827	-27,118	25,464	0,950	0,116	-1,271	1,502	0,869
Phocaeicola	F	-30,619	-89,108	27,870	0,293	-3,406	-10,123	3,311	0,308
Phocaeicola	G	-18,018	-58,368	22,332	0,376	0,939	-0,383	2,262	0,161
Prevotella	A	-5,772	-58,010	46,467	0,824	2,214	-4,551	8,979	0,511
Prevotella	B	10,157	-32,480	52,795	0,632	-0,058	-2,292	2,177	0,958
Prevotella	C	15,762	-20,568	52,091	0,391	1,219	-2,018	4,456	0,456
Prevotella	D	16,814	-7,062	40,690	0,163	1,346	-1,059	3,751	0,266
Prevotella	E	-16,780	-44,385	10,825	0,230	0,416	-3,693	4,526	0,841
Prevotella	F	-6,667	-48,280	34,947	0,745	0,000	-0,073	0,073	0,996
Prevotella	G	5,237	-22,027	32,501	0,703	0,501	-0,601	1,604	0,368
Roseburia	A	-12,069	-63,794	39,656	0,639	-0,087	-0,825	0,651	0,812
Roseburia	B	-11,104	-65,543	43,335	0,681	-0,589	-2,103	0,925	0,435
Roseburia	C	-28,801	-57,901	0,300	**0,052**	-0,547	-1,096	0,002	**0,051**
Roseburia	D	20,216	-24,215	64,647	0,364	-0,345	-1,953	1,263	0,667
Roseburia	E	-14,294	-45,100	16,513	0,359	-0,431	-1,639	0,776	0,480
Roseburia	F	-9,913	-67,695	47,869	0,728	-0,758	-3,245	1,728	0,537
Roseburia	G	-13,054	-48,534	22,425	0,466	-0,730	-2,059	0,600	0,278
Ruminococcus	A	-25,392	-68,617	17,833	0,242	-0,303	-2,393	1,787	0,771
Ruminococcus	B	-16,095	-67,941	35,751	0,533	0,394	-1,183	1,971	0,615
Ruminococcus	C	-40,581	-64,837	-16,325	**0,001**	-0,863	-1,403	-0,322	**0,002**
Ruminococcus	D	4,058	-34,816	42,931	0,834	0,003	-1,140	1,146	0,996
Ruminococcus	E	-17,470	-51,025	16,085	0,304	-0,321	-0,829	0,186	0,212
Ruminococcus	F	-16,300	-65,321	32,721	0,501	-0,009	-2,968	2,949	0,995
Ruminococcus	G	-6,668	-34,089	20,754	0,629	-0,296	-0,822	0,230	0,266
Streptococcus	A	-2,729	-59,306	53,848	0,923	-0,297	-1,205	0,610	0,511
Streptococcus	B	-64,831	-116,987	-12,675	**0,016**	-0,865	-2,119	0,389	0,170
Streptococcus	C	-22,527	-58,142	13,088	0,212	-0,240	-0,541	0,060	0,116
Streptococcus	D	-4,855	-48,534	38,824	0,824	-0,981	-2,574	0,612	0,221
Streptococcus	E	8,110	-27,689	43,909	0,654	-0,060	-0,394	0,274	0,723
Streptococcus	F	83,469	20,734	146,205	**0,011**	3,004	-2,584	8,592	0,280
Streptococcus	G	-42,529	-81,285	-3,773	**0,032**	-0,274	-0,604	0,057	0,104
Agathobacter	sum	11,230	-3,243	25,703	0,147	0,512	-0,224	1,247	0,174
Anaerobutyricum	sum	-13,668	-29,604	2,267	0,129	-0,100	-0,488	0,288	0,627
Anaerostipes	sum	41,198	22,962	59,435	**0,004**	1,477	0,598	2,355	**0,020**
Faecalibacterium	sum	16,711	4,330	29,092	**0,010**	1,418	0,477	2,359	**0,004**
Gemminger	sum	-5,669	-20,921	9,584	0,531	-0,085	-0,666	0,496	0,783
Oscillibacter	sum	-16,329	-35,850	3,191	0,164	-0,158	-0,319	0,003	0,105
Roseburia	sum	-13,192	-27,916	1,532	**0,084**	-0,531	-1,007	-0,056	**0,030**
Coprococcus	sum	-28,045	-44,425	-11,665	**0,021**	-0,694	-1,592	0,203	0,183
Akkermansia	sum	-6,901	-21,507	7,705	0,380	-0,007	-0,553	0,539	0,980
Bacteroides	sum	-3,485	-17,442	10,473	0,628	-0,179	-1,308	0,949	0,771
Dialister	sum	-4,228	-17,753	9,296	0,561	-0,120	-0,313	0,074	0,276
Phascolarctobacterium	sum	-7,830	-20,235	4,576	0,233	-0,659	-1,403	0,085	0,151
Phocaeicola	sum	-8,577	-22,433	5,278	0,230	-0,351	-1,227	0,524	0,432
Parabacteroides	sum	-19,816	-33,972	-5,659	**0,007**	-0,244	-0,608	0,120	0,190
Blautia	sum	-17,447	-31,545	-3,349	**0,053**	-1,151	-2,990	0,687	0,255
Mediterraneibacter	sum	-27,099	-48,231	-5,966	**0,045**	-0,209	-0,684	0,267	0,423
Bifidobacterium	sum	57,814	34,561	81,067	**0,008**	5,185	2,242	8,128	**0,015**
Dorea	sum	-6,491	-18,535	5,552	0,293	-0,233	-0,515	0,049	0,124
Fusicatenibacter	sum	6,080	-9,577	21,738	0,470	0,139	-0,155	0,433	0,371
Prevotella	sum	2,142	-11,854	16,138	0,767	0,840	-0,517	2,196	0,226
Ruminococcus	sum	-18,421	-31,770	-5,073	**0,008**	-0,307	-0,696	0,082	0,126
Streptococcus	sum	-8,981	-40,165	22,202	0,599	-0,049	-0,850	0,752	0,911

Supplementary Table 4. Response of bacteria exhibiting SCFA pathways to RS treatment compared with controls. The estimate along with the 95 % confidence interval is given. Results based on both responses relative to pre-intervention and relative abundance changes are given for all studies and the pooled analysis.

		relative response				*relative abundance change*			
**pathway**	**dataset**	**estimate**	**0,025**	**0,975**	**p**	**estimate**	**0,025**	**0,975**	**p**
AcCoA	C_m	1,262	-11,417	13,941	0,843	0,455	-1,560	2,470	0,655
AcCoA	C_p	5,618	-9,780	21,016	0,470	1,379	-1,171	3,928	0,285
AcCoA	I	32,358	-6,576	71,292	0,101	11,492	-0,084	23,068	**0,052**
AcCoA	J	12,708	-27,010	52,427	0,519	2,605	-8,696	13,905	0,642
AcCoA	K_t	5,015	-13,644	23,674	0,579	1,001	-4,716	6,718	0,717
AcCoA	K_m	18,552	1,025	36,079	**0,039**	5,119	0,750	9,488	**0,024**
AcCoA	K_p	-1,078	-19,691	17,535	0,905	-0,026	-5,035	4,983	0,991
AcCoA	L	-17,556	-59,083	23,971	0,386	-3,067	-9,679	3,545	0,343
But	C_m	5,483	-7,787	18,752	0,413	0,845	-1,011	2,700	0,368
But	C_p	9,723	-5,988	25,434	0,222	1,899	-0,460	4,258	0,113
But	I	1,822	-41,281	44,924	0,932	0,352	-7,027	7,731	0,924
But	J	14,226	-27,599	56,052	0,493	2,584	-7,287	12,454	0,598
But	K_t	15,628	-15,019	46,275	0,298	2,959	-3,290	9,208	0,333
But	K_m	27,988	8,770	47,206	**0,007**	6,259	2,663	9,855	**0,002**
But	K_p	-0,253	-19,366	18,860	0,978	0,134	-3,994	4,262	0,946
But	L	-1,637	-42,085	38,811	0,933	-0,915	-7,621	5,791	0,778
Buk	C_m	-19,511	-48,806	9,783	0,189	-0,310	-0,794	0,174	0,206
Buk	C_p	-34,511	-65,018	-4,003	**0,027**	-0,353	-1,039	0,333	0,309
Buk	I	43,308	-5,151	91,766	**0,078**	9,231	-0,240	18,702	**0,056**
Buk	J	-1,160	-49,297	46,977	0,961	0,161	-2,699	3,021	0,909
Buk	K_t	-13,757	-65,060	37,546	0,580	0,067	-3,351	3,485	0,968
Buk	K_m	-22,738	-62,681	17,205	0,247	-0,987	-3,136	1,162	0,347
Buk	K_p	-25,684	-59,128	7,760	0,124	-1,017	-2,632	0,598	0,202
Buk	L	-4,256	-79,762	71,250	0,907	-0,696	-4,682	3,290	0,718
Suc	C_m	-3,578	-15,366	8,209	0,547	-1,668	-5,338	2,001	0,368
Suc	C_p	-5,669	-20,736	9,398	0,456	-2,872	-7,390	1,646	0,210
Suc	I	-3,336	-52,647	45,976	0,892	-3,406	-15,891	9,079	0,584
Suc	J	-4,416	-37,886	29,053	0,790	1,472	-14,440	17,385	0,852
Suc	K_t	6,708	-24,646	38,062	0,658	0,631	-4,533	5,795	0,800
Suc	K_m	-46,918	-80,240	-13,596	**0,008**	-7,307	-12,151	-2,463	**0,005**
Suc	K_p	-0,935	-37,561	35,691	0,958	-0,956	-6,525	4,613	0,723
Suc	L	11,588	-68,801	91,977	0,765	-0,785	-9,842	8,272	0,858
Pdiol	C_m	-41,066	-64,974	-17,157	**0,001**	-1,505	-2,412	-0,597	**0,001**
Pdiol	C_p	-35,196	-59,882	-10,511	**0,006**	-1,461	-2,651	-0,271	**0,017**
Pdiol	I	-10,444	-58,640	37,752	0,663	-1,756	-4,725	1,213	0,239
Pdiol	J	-29,919	-77,999	18,160	0,214	-1,081	-2,923	0,760	0,241
Pdiol	K_t	-24,137	-58,364	10,090	0,156	-1,836	-5,129	1,457	0,257
Pdiol	K_m	-15,125	-41,702	11,452	0,247	-1,512	-4,196	1,172	0,252
Pdiol	K_p	16,054	-19,915	52,023	0,361	1,300	-2,492	5,092	0,481
Pdiol	L	11,337	-45,668	68,342	0,681	1,633	-3,443	6,709	0,508
AcCoA	sum	8,532	−2,386	19,450	0,180	2,465	−0,601	5,532	0,165
But	sum	8,700	−0,970	18,369	**0,092**	1,625	−0,195	3,445	0,101
Buk	sum	-9,058	−29,095	10,978	0,399	0,978	−0,923	2,880	0,333
Suc	sum	-5,056	−15,253	5,142	0,332	-1,966	−4,704	0,773	0,161
Pdiol	sum	-23,192	-39,334	-7,050	**0,032**	-1,060	-1,800	-0,320	**0,010**

Supplementary Table 5. Response of individual taxa to RS treatment compared with controls. The estimate along with the 95 % confidence interval is given. Results based on both responses relative to pre-intervention and relative abundance changes are given for all studies and the pooled analysis.

		relative response				*relative abundance change*			
**taxon**	**dataset**	**estimate**	**0,025**	**0,975**	**p**	**estimate**	**0,025**	**0,975**	**p**
Agathobacter	C_maz	20,964	-7,436	49,363	0,146	0,706	0,029	1,384	**0,041**
Agathobacter	C_pot	10,181	-18,758	39,119	0,486	1,180	0,225	2,135	**0,016**
Agathobacter	I	7,649	-45,233	60,531	0,771	1,110	-0,417	2,636	0,149
Agathobacter	J	-5,367	-31,646	20,913	0,680	-0,006	-0,014	0,002	0,142
Agathobacter	K_tap	61,031	-1,778	123,840	**0,056**	3,662	-1,344	8,668	0,142
Agathobacter	K_maz	106,312	63,546	149,079	**0,000**	6,576	3,029	10,122	**0,001**
Agathobacter	K_pot	42,110	-14,698	98,917	0,137	1,909	-1,458	5,276	0,249
Agathobacter	L	21,615	-54,333	97,564	0,557	-0,364	-3,088	2,359	0,782
Akkermansia	C_maz	-4,458	-34,990	26,075	0,772	0,026	-0,580	0,632	0,932
Akkermansia	C_pot	-1,194	-27,806	25,417	0,929	-0,240	-0,860	0,379	0,442
Akkermansia	I	-43,080	-100,487	14,327	0,137	-3,141	-8,710	2,427	0,261
Akkermansia	J	22,173	-29,865	74,212	0,392	0,100	-0,208	0,408	0,512
Akkermansia	K_tap	-71,844	-146,979	3,290	**0,060**	-0,514	-1,474	0,446	0,275
Akkermansia	K_maz	-15,604	-96,976	65,768	0,692	-1,136	-2,828	0,556	0,175
Akkermansia	K_pot	-24,290	-97,359	48,778	0,494	-0,368	-1,333	0,597	0,433
Akkermansia	L	-3,060	-63,516	57,396	0,916	-0,356	-0,972	0,261	0,241
Anaerobutyricum	C_maz	-29,429	-57,779	-1,080	**0,042**	-0,152	-0,325	0,021	**0,084**
Anaerobutyricum	C_pot	-9,458	-40,497	21,581	0,546	-0,067	-0,257	0,122	0,481
Anaerobutyricum	I	-10,678	-63,113	41,757	0,682	-0,497	-1,262	0,267	0,196
Anaerobutyricum	J	-23,172	-79,734	33,389	0,410	-0,156	-0,614	0,302	0,493
Anaerobutyricum	K_tap	-20,085	-75,610	35,440	0,457	-0,067	-0,826	0,692	0,855
Anaerobutyricum	K_maz	2,421	-47,636	52,477	0,920	0,242	-0,423	0,906	0,455
Anaerobutyricum	K_pot	-6,274	-64,883	52,336	0,825	-0,067	-0,922	0,789	0,872
Anaerobutyricum	L	-24,577	-86,214	37,061	0,413	-0,269	-1,114	0,575	0,511
Anaerostipes	C_maz	-27,601	-59,268	4,067	**0,087**	-0,335	-0,666	-0,003	**0,048**
Anaerostipes	C_pot	-30,871	-61,400	-0,342	**0,048**	-0,311	-0,692	0,069	0,108
Anaerostipes	I	-34,991	-93,207	23,224	0,231	-0,178	-0,485	0,129	0,248
Anaerostipes	J	-33,002	-87,703	21,699	0,228	-0,305	-0,827	0,217	0,243
Anaerostipes	K_tap	-23,022	-77,280	31,236	0,384	-0,495	-1,577	0,588	0,350
Anaerostipes	K_maz	-52,327	-101,806	-2,848	**0,039**	-0,512	-1,316	0,292	0,197
Anaerostipes	K_pot	-22,420	-67,866	23,026	0,314	-0,474	-1,304	0,355	0,245
Anaerostipes	L	-33,182	-98,357	31,993	0,299	0,238	-0,914	1,391	0,669
Bacteroides	C_maz	-4,911	-23,672	13,849	0,604	-1,209	-3,153	0,734	0,219
Bacteroides	C_pot	-0,838	-20,315	18,638	0,932	-0,582	-2,641	1,477	0,575
Bacteroides	I	27,890	-27,092	82,872	0,311	-0,115	-2,996	2,766	0,936
Bacteroides	J	-12,290	-55,924	31,344	0,570	-0,131	-7,355	7,093	0,971
Bacteroides	K_tap	-20,686	-77,491	36,120	0,454	-0,909	-4,937	3,119	0,641
Bacteroides	K_maz	-46,275	-94,857	2,306	**0,061**	-1,457	-4,735	1,820	0,363
Bacteroides	K_pot	-13,531	-63,793	36,730	0,579	0,120	-3,648	3,888	0,947
Bacteroides	L	-25,379	-108,428	57,669	0,529	-2,880	-5,983	0,222	**0,067**
Bifidobacterium	C_maz	-16,652	-47,572	14,269	0,287	-0,165	-0,547	0,217	0,393
Bifidobacterium	C_pot	54,590	24,022	85,159	**0,001**	3,719	2,258	5,180	**0,000**
Bifidobacterium	I	-22,167	-76,014	31,680	0,410	-2,507	-4,723	-0,290	**0,028**
Bifidobacterium	J	0,096	-60,588	60,780	0,997	0,001	-0,458	0,460	0,996
Bifidobacterium	K_tap	28,358	-34,063	90,779	0,352	4,605	-0,971	10,181	**0,100**
Bifidobacterium	K_maz	9,919	-47,709	67,548	0,722	4,130	-0,819	9,078	**0,097**
Bifidobacterium	K_pot	-15,203	-86,805	56,398	0,661	1,769	-2,991	6,529	0,445
Bifidobacterium	L	13,036	-41,247	67,318	0,620	-0,004	-0,547	0,539	0,987
Blautia	C_maz	-28,839	-53,482	-4,196	**0,022**	-0,754	-1,398	-0,110	**0,022**
Blautia	C_pot	-35,158	-62,129	-8,188	**0,011**	-1,042	-1,827	-0,256	**0,010**
Blautia	I	-29,435	-72,301	13,431	0,173	-1,484	-3,240	0,273	**0,095**
Blautia	J	-29,721	-78,912	19,470	0,227	-0,637	-1,715	0,440	0,237
Blautia	K_tap	-35,681	-83,125	11,764	0,132	-1,272	-3,336	0,793	0,212
Blautia	K_maz	-35,665	-79,261	7,932	0,103	-1,308	-3,290	0,673	0,182
Blautia	K_pot	11,307	-40,031	62,645	0,649	0,122	-2,536	2,780	0,924
Blautia	L	19,284	-47,067	85,635	0,549	1,402	-2,727	5,531	0,485

Coprococcus	C_maz	-24,503	-52,310	3,305	**0,083**	-0,147	-0,343	0,049	0,139
Coprococcus	C_pot	-41,997	-67,410	-16,584	**0,001**	-0,277	-0,468	-0,087	**0,005**
Coprococcus	I	-4,775	-56,386	46,835	0,852	0,640	-0,222	1,503	0,141
Coprococcus	J	-3,004	-58,080	52,072	0,912	-0,067	-0,392	0,258	0,678
Coprococcus	K_tap	-28,836	-95,817	38,146	0,378	0,197	-0,712	1,106	0,654
Coprococcus	K_maz	-45,923	-92,995	1,149	**0,055**	-0,553	-1,448	0,343	0,211
Coprococcus	K_pot	-41,361	-97,883	15,161	0,142	-0,636	-1,478	0,207	0,131
Coprococcus	L	-22,772	-97,656	52,113	0,531	0,024	-1,233	1,280	0,969
Dialister	C_maz	-12,319	-41,064	16,426	0,396	-0,058	-0,280	0,165	0,608
Dialister	C_pot	-14,149	-44,324	16,027	0,354	-0,049	-0,263	0,166	0,653
Dialister	I	14,788	-29,343	58,920	0,502	1,926	-7,860	11,712	0,693
Dialister	J	7,252	-44,333	58,838	0,776	0,062	-0,218	0,342	0,654
Dialister	K_tap	26,134	-36,732	88,999	0,394	0,026	-0,016	0,068	0,215
Dialister	K_maz	-11,533	-80,636	57,570	0,730	0,010	-0,047	0,067	0,707
Dialister	K_pot	-34,296	-95,219	26,628	0,252	-0,021	-0,076	0,033	0,425
Dialister	L	-12,241	-61,253	36,770	0,606	-0,318	-0,870	0,234	0,241
Dorea	C_maz	-28,934	-56,412	-1,455	**0,039**	-0,236	-0,437	-0,035	**0,022**
Dorea	C_pot	-36,399	-63,521	-9,276	**0,009**	-0,228	-0,417	-0,039	**0,019**
Dorea	I	-17,164	-70,268	35,939	0,517	0,391	-1,162	1,944	0,613
Dorea	J	-1,957	-58,013	54,100	0,944	-0,140	-0,402	0,122	0,285
Dorea	K_tap	-39,421	-95,467	16,625	0,157	-0,703	-1,718	0,312	0,163
Dorea	K_maz	-35,683	-94,790	23,424	0,221	-0,737	-1,792	0,318	0,160
Dorea	K_pot	-7,632	-64,102	48,838	0,780	-0,232	-1,327	0,864	0,662
Dorea	L	-34,994	-101,336	31,349	0,282	-1,773	-3,846	0,300	**0,089**
Faecalibacterium	C_maz	5,616	-14,499	25,731	0,580	0,315	-0,854	1,484	0,593
Faecalibacterium	C_pot	2,639	-17,983	23,261	0,800	0,417	-0,873	1,707	0,522
Faecalibacterium	I	-26,707	-75,263	21,849	0,273	-1,164	-6,668	4,340	0,671
Faecalibacterium	J	19,371	-37,962	76,705	0,496	0,659	-0,953	2,270	0,411
Faecalibacterium	K_tap	11,410	-36,507	59,328	0,623	1,030	-1,668	3,728	0,433
Faecalibacterium	K_maz	-0,291	-35,745	35,162	0,986	0,104	-2,331	2,538	0,930
Faecalibacterium	K_pot	-2,645	-45,842	40,553	0,899	0,134	-2,669	2,936	0,921
Faecalibacterium	L	-3,709	-80,286	72,867	0,920	-0,767	-4,653	3,118	0,683
Fusicatenibacter	C_maz	-14,367	-42,092	13,357	0,306	-0,218	-0,432	-0,004	**0,046**
Fusicatenibacter	C_pot	-19,686	-47,487	8,115	0,163	-0,072	-0,292	0,148	0,517
Fusicatenibacter	I	66,616	12,454	120,778	**0,017**	3,454	-2,014	8,922	0,209
Fusicatenibacter	J	19,015	-35,240	73,270	0,480	0,003	-0,577	0,584	0,990
Fusicatenibacter	K_tap	-24,100	-83,243	35,043	0,403	-0,725	-2,138	0,687	0,295
Fusicatenibacter	K_maz	-56,609	-110,616	-2,602	**0,041**	-1,528	-3,350	0,295	**0,095**
Fusicatenibacter	K_pot	-41,155	-94,775	12,466	0,124	-1,128	-2,463	0,207	**0,093**
Fusicatenibacter	L	-37,754	-100,632	25,124	0,223	-0,554	-1,188	0,080	**0,083**
Gemminger	C_maz	6,745	-20,465	33,956	0,623	0,037	-0,162	0,235	0,714
Gemminger	C_pot	0,084	-29,695	29,863	0,996	-0,016	-0,314	0,283	0,918
Gemminger	I	15,416	-37,864	68,696	0,562	2,582	-3,615	8,780	0,404
Gemminger	J	-6,511	-57,364	44,341	0,796	0,097	-0,569	0,763	0,768
Gemminger	K_tap	-22,492	-94,909	49,925	0,522	-0,023	-0,527	0,481	0,925
Gemminger	K_maz	-41,735	-94,713	11,243	0,115	-0,114	-0,438	0,209	0,467
Gemminger	K_pot	-59,490	-112,637	-6,343	**0,030**	-0,131	-0,459	0,197	0,411
Gemminger	L	-34,482	-92,632	23,668	0,229	-0,377	-2,151	1,396	0,660
Mediterraneibacter	C_maz	-22,206	-50,235	5,822	0,119	-0,189	-0,368	-0,010	**0,038**
Mediterraneibacter	C_pot	-26,668	-54,392	1,056	**0,059**	-0,184	-0,361	-0,008	**0,041**
Mediterraneibacter	I	5,614	-49,106	60,333	0,837	1,006	0,002	2,010	**0,050**
Mediterraneibacter	J	-23,843	-82,043	34,356	0,410	-0,171	-0,421	0,079	0,173
Mediterraneibacter	K_tap	-23,923	-82,076	34,229	0,399	0,247	-0,758	1,252	0,612
Mediterraneibacter	K_maz	-11,305	-77,941	55,330	0,726	0,297	-0,780	1,375	0,569
Mediterraneibacter	K_pot	55,168	-4,889	115,225	**0,070**	1,078	-0,079	2,236	**0,066**
Mediterraneibacter	L	-37,245	-98,057	23,567	0,214	-1,036	-2,466	0,394	0,145
Oscillibacter	C_maz	-4,757	-32,248	22,733	0,731	-0,136	-0,358	0,086	0,225
Oscillibacter	C_pot	-3,309	-31,997	25,379	0,819	-0,071	-0,218	0,075	0,336
Oscillibacter	I	26,411	-28,100	80,921	0,333	1,036	-0,740	2,812	0,245
Oscillibacter	J	-42,794	-96,436	10,848	0,114	-2,680	-7,229	1,869	0,239
Oscillibacter	K_tap	-0,166	-69,324	68,992	0,996	-0,123	-0,587	0,341	0,585
Oscillibacter	K_maz	13,808	-44,237	71,854	0,623	0,049	-0,199	0,297	0,682
Oscillibacter	K_pot	7,398	-44,788	59,583	0,769	0,119	-0,088	0,327	0,242
Oscillibacter	L	26,674	-46,639	99,987	0,455	1,784	-1,608	5,176	0,284

Parabacteroides	C_maz	-9,471	-36,271	17,329	0,484	-0,323	-0,887	0,240	0,257
Parabacteroides	C_pot	-25,108	-51,882	1,666	**0,066**	-0,346	-0,764	0,071	0,102
Parabacteroides	I	78,095	25,046	131,145	**0,005**	0,204	0,022	0,385	**0,029**
Parabacteroides	J	-33,495	-84,430	17,440	0,190	-0,868	-2,086	0,350	0,156
Parabacteroides	K_tap	45,061	-7,742	97,864	**0,090**	4,173	1,834	6,513	**0,001**
Parabacteroides	K_maz	-69,572	-136,230	-2,913	**0,042**	-1,688	-2,970	-0,405	**0,013**
Parabacteroides	K_pot	12,030	-46,676	70,736	0,672	-0,150	-1,428	1,128	0,808
Parabacteroides	L	16,316	-71,077	103,709	0,699	0,049	-1,958	2,056	0,960
Phascolarctobacterium	C_maz	13,719	-10,233	37,672	0,258	-0,351	-0,718	0,017	**0,061**
Phascolarctobacterium	C_pot	-7,579	-29,229	14,072	0,488	-0,206	-0,542	0,129	0,224
Phascolarctobacterium	I	18,847	-27,432	65,127	0,415	0,974	-0,140	2,087	**0,085**
Phascolarctobacterium	J	10,806	-31,501	53,113	0,606	-0,185	-1,031	0,660	0,658
Phascolarctobacterium	K_tap	-20,338	-67,466	26,790	0,377	-0,228	-0,720	0,263	0,342
Phascolarctobacterium	K_maz	-17,726	-74,094	38,641	0,517	-0,161	-0,673	0,350	0,515
Phascolarctobacterium	K_pot	-7,295	-52,151	37,562	0,737	0,283	-0,272	0,838	0,299
Phascolarctobacterium	L	24,557	-37,001	86,116	0,413	0,618	-0,501	1,736	0,261
Phocaeicola	C_maz	-8,505	-26,886	9,875	0,360	-0,154	-3,170	2,862	0,919
Phocaeicola	C_pot	-9,917	-29,792	9,959	0,324	-1,012	-3,979	1,955	0,499
Phocaeicola	I	19,889	-39,044	78,822	0,499	-0,091	-2,744	2,563	0,945
Phocaeicola	J	-1,109	-46,342	44,123	0,960	0,470	-8,656	9,596	0,917
Phocaeicola	K_tap	-32,184	-92,382	28,013	0,276	-1,526	-3,992	0,940	0,210
Phocaeicola	K_maz	-54,055	-110,138	2,029	**0,058**	-2,402	-5,070	0,266	**0,075**
Phocaeicola	K_pot	-44,741	-106,202	16,720	0,144	-2,095	-4,362	0,171	**0,068**
Phocaeicola	L	8,729	-78,010	95,468	0,835	1,242	-2,439	4,923	0,487
Prevotella	C_maz	19,568	-15,625	54,760	0,272	1,943	-0,992	4,877	0,192
Prevotella	C_pot	6,459	-28,068	40,986	0,711	0,703	-2,022	3,428	0,609
Prevotella	I	6,172	-46,511	58,855	0,814	-0,249	-3,763	3,266	0,887
Prevotella	J	-13,689	-68,943	41,564	0,617	-4,630	-10,833	1,574	0,138
Prevotella	K_tap	46,991	-23,885	117,867	0,181	1,866	-0,059	3,791	**0,057**
Prevotella	K_maz	30,766	-31,406	92,939	0,312	0,958	-0,577	2,492	0,206
Prevotella	K_pot	9,782	-62,828	82,393	0,780	2,071	-0,798	4,940	0,147
Prevotella	L	-24,158	-102,736	54,420	0,526	0,272	-1,481	2,026	0,748
Roseburia	C_maz	12,799	-18,559	44,156	0,419	0,351	-0,294	0,996	0,282
Roseburia	C_pot	2,544	-30,993	36,080	0,880	0,285	-0,372	0,942	0,391
Roseburia	I	12,666	-41,415	66,748	0,638	0,629	-0,341	1,600	0,197
Roseburia	J	14,414	-43,211	72,038	0,614	1,353	-1,722	4,428	0,377
Roseburia	K_tap	27,035	-46,524	100,594	0,450	-0,202	-4,321	3,917	0,919
Roseburia	K_maz	-12,890	-83,669	57,889	0,706	0,468	-1,793	2,728	0,669
Roseburia	K_pot	5,199	-63,134	73,531	0,875	-0,237	-2,392	1,918	0,820
Roseburia	L	11,673	-69,209	92,556	0,765	0,215	-0,729	1,159	0,638
Ruminococcus	C_maz	15,285	-12,334	42,903	0,274	1,449	0,585	2,313	**0,001**
Ruminococcus	C_pot	-37,251	-64,245	-10,258	**0,007**	-0,366	-1,235	0,504	0,405
Ruminococcus	I	-10,524	-63,954	42,907	0,692	-0,309	-1,903	1,285	0,697
Ruminococcus	J	-2,862	-58,293	52,569	0,917	-0,005	-0,645	0,636	0,988
Ruminococcus	K_tap	-10,916	-83,349	61,517	0,755	-0,172	-1,089	0,745	0,698
Ruminococcus	K_maz	-73,046	-140,337	-5,754	**0,035**	-0,739	-1,599	0,121	**0,088**
Ruminococcus	K_pot	-17,447	-85,122	50,227	0,595	-0,218	-1,044	0,608	0,586
Ruminococcus	L	45,176	-21,514	111,866	0,172	6,754	-3,919	17,428	0,200
Streptococcus	C_maz	-32,612	-69,186	3,963	**0,080**	-0,142	-0,304	0,021	**0,087**
Streptococcus	C_pot	-27,775	-63,681	8,131	0,128	-0,052	-0,257	0,153	0,616
Streptococcus	I	-60,796	-109,836	-11,755	**0,016**	-2,787	-5,367	-0,207	**0,035**
Streptococcus	J	-11,323	-68,929	46,283	0,692	0,007	-0,088	0,103	0,875
Streptococcus	K_tap	-26,003	-103,231	51,224	0,488	-1,409	-4,105	1,287	0,287
Streptococcus	K_maz	-14,377	-87,839	59,085	0,686	-0,853	-2,188	0,482	0,196
Streptococcus	K_pot	-32,849	-101,319	35,622	0,327	-0,964	-2,266	0,338	0,137
Streptococcus	L	-41,241	-126,131	43,648	0,321	-3,053	-6,611	0,506	**0,088**

Agathobacter	sum	26,204	2,867	49,540	**0,081**	1,732	0,307	3,158	**0,046**
Anaerobutyricum	sum	-17,129	-32,441	-1,817	**0,032**	-0,119	-0,283	0,044	0,173
Anaerostipes	sum	-31,585	-47,141	-16,028	**0,000**	-0,304	-0,485	-0,122	**0,001**
Faecalibacterium	sum	1,151	-12,046	14,347	0,866	0,125	-0,799	1,048	0,793
Gemminger	sum	-7,519	-23,015	7,978	0,365	0,300	-0,461	1,060	0,441
Oscillibacter	sum	1,673	-17,737	21,082	0,887	-0,047	-1,007	0,913	0,926
Roseburia	sum	9,411	-7,474	26,297	0,277	0,438	-0,112	0,989	0,137
Coprococcus	sum	-26,883	-41,384	-12,382	**0,000**	-0,085	-0,366	0,195	0,574
Akkermansia	sum	-13,480	-30,712	3,752	0,172	-0,647	-1,517	0,223	0,191
Bacteroides	sum	-6,678	-20,628	7,272	0,404	-0,807	-1,960	0,346	0,171
Dialister	sum	-6,305	-21,235	8,625	0,411	0,233	-1,028	1,494	0,720
Phascolarctobacterium	sum	4,429	-8,445	17,303	0,506	0,059	-0,272	0,389	0,737
Phocaeicola	sum	-10,079	-23,538	3,379	0,280	-0,568	-2,045	0,910	0,452
Parabacteroides	sum	2,836	-29,163	34,836	0,867	0,170	-0,990	1,330	0,783
Blautia	sum	-26,022	-39,555	-12,489	**0,001**	-0,730	-1,260	-0,201	**0,025**
Mediterraneibacter	sum	-14,371	-33,216	4,475	0,179	0,102	-0,354	0,558	0,676
Bifidobacterium	sum	8,314	-14,783	31,412	0,497	1,363	-0,404	3,129	0,174
Dorea	sum	-26,815	-41,542	-12,088	**0,000**	-0,406	-0,786	-0,026	**0,103**
Fusicatenibacter	sum	-11,178	-38,680	16,323	0,454	0,116	-0,620	0,851	0,758
Prevotella	sum	10,243	-6,897	27,383	0,242	0,317	-1,353	1,987	0,720
Ruminococcus	sum	-11,742	-32,264	8,779	0,290	0,760	-0,673	2,193	0,341
Streptococcus	sum	-31,473	-49,261	-13,684	**0,001**	-1,056	-1,883	-0,229	**0,040**

Supplementary Table 6. Response of bacteria exhibiting SCFA pathways to AXOS treatment compared with controls. The estimate along with the 95 % confidence interval is given. Results based on both responses relative to pre-intervention and relative abundance changes are given for all studies and the pooled analysis.

		relative response				*relative abundance change*			
**pathway**	**dataset**	**estimate**	**0,025**	**0,975**	**p**	**estimate**	**0,025**	**0,975**	**p**
AcCoA	M	-16,417	-29,985	-2,848	**0,019**	-3,626	-6,603	-0,649	**0,018**
AcCoA	N	-27,440	-64,153	9,273	0,138	-6,730	-14,058	0,598	**0,071**
But	M	-15,648	-32,217	0,921	**0,063**	-2,597	-5,176	-0,018	**0,048**
But	N	-29,152	-66,331	8,027	0,121	-5,418	-11,423	0,587	**0,076**
Buk	M	-19,414	-46,965	8,137	0,162	-0,458	-1,618	0,703	0,430
Buk	N	-44,849	-81,742	-7,957	**0,019**	-1,601	-3,279	0,077	**0,061**
Suc	M	-22,798	-67,159	21,564	0,305	-3,729	-7,065	-0,392	**0,030**
Suc	N	-17,955	-66,349	30,438	0,457	0,350	-5,479	6,179	0,904
Pdiol	M	-49,384	-77,586	-21,182	**0,001**	-1,808	-2,927	-0,688	**0,002**
Pdiol	N	-49,002	-91,208	-6,797	**0,024**	-8,147	-14,282	-2,012	**0,011**
AcCoA	sum	-22,059	-43,392	-0,727	0,163	-5,201	-9,993	-0,410	0,199
But	sum	-22,546	-45,742	0,650	0,203	-4,029	-8,132	0,074	0,237
Buk	sum	-32,271	-65,084	0,543	0,264	-1,036	-2,497	0,425	0,363
Suc	sum	-21,350	-52,761	10,061	0,187	-1,754	-4,920	1,411	0,281
Pdiol	sum	-49,958	-74,151	-25,765	**0,000**	-4,908	-9,374	-0,441	0,186

Supplementary Table 7. Response of individual taxa to AXOS treatment compared with controls. The estimate along with the 95 % confidence interval is given. Results based on both responses relative to pre-intervention and relative abundance changes are given for all studies and the pooled analysis.

		relative response				*relative abundance change*			
**taxon**	**dataset**	**estimate**	**0,025**	**0,975**	**p**	**estimate**	**0,025**	**0,975**	**p**
Agathobacter	M	9,181	-36,856	55,218	0,689	-0,045	-0,752	0,663	0,899
Agathobacter	N	-37,426	-93,634	18,782	0,185	0,010	-0,161	0,180	0,910
Akkermansia	M	-4,370	-34,716	25,975	0,772	0,014	-0,023	0,051	0,444
Akkermansia	N	12,677	-47,199	72,554	0,670	1,091	-2,074	4,256	0,489
Anaerobutyricum	M	-17,833	-55,391	19,724	0,343	-0,200	-0,487	0,087	0,167
Anaerobutyricum	N	-40,787	-85,793	4,218	**0,074**	-0,862	-2,154	0,430	0,185
Anaerostipes	M	13,111	-29,913	56,136	0,541	-0,105	-0,823	0,612	0,768
Anaerostipes	N	15,226	-34,841	65,292	0,542	0,077	-1,133	1,287	0,898
Bacteroides	M	-16,999	-44,988	10,989	0,226	1,055	-0,980	3,090	0,301
Bacteroides	N	2,648	-31,478	36,775	0,876	0,455	-0,852	1,762	0,485
Bifidobacterium	M	124,422	92,389	156,455	**0,000**	9,854	6,972	12,737	**0,000**
Bifidobacterium	N	20,812	-19,593	61,216	0,303	11,983	-0,949	24,915	**0,068**
Blautia	M	-42,088	-78,916	-5,260	**0,026**	-0,568	-1,124	-0,012	**0,046**
Blautia	N	-47,169	-93,077	-1,261	**0,044**	-5,772	-11,096	-0,448	**0,034**
Coprococcus	M	-13,509	-60,557	33,539	0,564	-0,004	-0,242	0,235	0,976
Coprococcus	N	-34,396	-77,621	8,830	0,115	-0,857	-1,980	0,266	0,130
Dialister	M	-4,658	-36,627	27,310	0,770	0,092	-0,391	0,574	0,703
Dialister	N	-14,518	-70,373	41,337	0,602	-0,884	-2,395	0,627	0,244
Dorea	M	-64,843	-101,394	-28,292	**0,001**	-0,302	-0,488	-0,115	**0,002**
Dorea	N	-51,721	-98,854	-4,588	**0,032**	-1,314	-2,314	-0,315	**0,011**
Faecalibacterium	M	-16,454	-43,905	10,997	0,232	-1,559	-3,843	0,725	0,175
Faecalibacterium	N	-0,413	-47,848	47,022	0,986	-1,817	-4,574	0,941	0,190
Fusicatenibacter	M	-0,611	-43,435	42,214	0,977	-0,037	-0,607	0,534	0,897
Fusicatenibacter	N	-36,854	-85,099	11,390	0,130	-0,504	-1,335	0,327	0,227
Gemminger	M	1,023	-13,365	15,410	0,886	-0,006	-0,033	0,021	0,635
Gemminger	N	3,613	-47,504	54,730	0,887	0,046	-0,724	0,815	0,905
Mediterraneibacter	M	-35,747	-72,616	1,121	**0,057**	-0,353	-0,670	-0,037	**0,029**
Mediterraneibacter	N	-35,393	-79,377	8,592	0,112	-0,838	-1,349	-0,327	**0,002**
Oscillibacter	M	-57,821	-94,935	-20,707	**0,003**	-0,461	-0,873	-0,048	**0,030**
Oscillibacter	N	-14,939	-56,743	26,866	0,474	0,071	-0,729	0,870	0,859
Parabacteroides	M	-60,423	-99,537	-21,309	**0,003**	-0,381	-0,765	0,003	**0,052**
Parabacteroides	N	-0,955	-40,281	38,372	0,961	0,448	-0,427	1,323	0,306
Phascolarctobacterium	M	-85,743	-116,275	-55,211	**0,000**	-0,576	-0,895	-0,258	**0,001**
Phascolarctobacterium	N	-1,335	-46,581	43,911	0,953	0,094	-0,319	0,506	0,649
Phocaeicola	M	-34,135	-66,460	-1,810	**0,039**	-2,304	-4,359	-0,250	**0,029**
Phocaeicola	N	-18,687	-57,920	20,547	0,341	-0,034	-1,546	1,478	0,964
Prevotella	M	14,435	-19,173	48,043	0,390	5,655	1,387	9,923	**0,011**
Prevotella	N	-29,176	-84,945	26,594	0,296	1,514	-2,755	5,784	0,477
Roseburia	M	6,660	-40,567	53,888	0,777	0,126	-0,611	0,863	0,731
Roseburia	N	-61,366	-110,685	-12,047	**0,016**	-2,260	-4,133	-0,388	**0,019**
Ruminococcus	M	2,296	-32,427	37,020	0,894	0,026	-0,376	0,428	0,897
Ruminococcus	N	-29,264	-72,545	14,018	0,179	-1,839	-4,017	0,338	**0,095**
Streptococcus	M	-29,709	-76,870	17,452	0,210	-0,248	-1,075	0,579	0,547
Streptococcus	N	-12,907	-70,282	44,468	0,651	0,837	-0,846	2,521	0,320
Agathobacter	sum	-14,408	-65,965	37,149	0,670	-0,021	-0,374	0,331	0,906
Anaerobutyricum	sum	-29,839	-63,966	4,289	0,266	-0,519	-1,206	0,169	0,349
Anaerostipes	sum	13,549	-18,125	45,224	0,406	-0,034	-0,702	0,634	0,920
Faecalibacterium	sum	-8,721	-38,780	21,339	0,620	-1,706	-3,438	0,026	**0,068**
Gemminger	sum	1,630	-23,523	26,783	0,899	0,012	-0,354	0,377	0,952
Oscillibacter	sum	-36,684	-84,451	11,083	0,357	-0,200	-0,845	0,444	0,635
Roseburia	sum	-27,628	-78,477	23,221	0,389	-1,052	-2,427	0,323	0,281
Coprococcus	sum	-24,407	-59,505	10,690	0,290	-0,435	-1,376	0,507	0,520
Akkermansia	sum	5,220	-26,383	36,823	0,747	0,588	-0,889	2,064	0,438
Bacteroides	sum	-7,302	-35,702	21,099	0,681	0,735	-0,432	1,901	0,220
Dialister	sum	-10,107	-40,603	20,390	0,580	-0,405	-1,189	0,380	0,380
Phascolarctobacterium	sum	-43,604	-126,333	39,124	0,490	-0,241	-0,904	0,421	0,650
Phocaeicola	sum	-26,671	-54,958	1,616	0,195	-1,194	-2,776	0,389	0,273
Parabacteroides	sum	-30,911	-93,320	31,499	0,506	0,028	-0,810	0,866	0,985
Blautia	sum	-45,194	-73,226	-17,161	**0,002**	-3,141	-6,741	0,459	0,230
Mediterraneibacter	sum	-35,960	-63,458	-8,462	**0,012**	-0,598	-1,119	-0,076	0,252
Bifidobacterium	sum	72,753	-29,758	175,264	0,397	10,962	4,374	17,551	**0,021**
Dorea	sum	-59,262	-87,754	-30,769	**0,000**	-0,808	-1,524	-0,092	0,153
Fusicatenibacter	sum	-19,366	-54,525	15,793	0,341	-0,277	-0,860	0,307	0,441
Prevotella	sum	-7,893	-47,834	32,048	0,725	3,560	-1,084	8,205	0,351
Ruminococcus	sum	-13,723	-52,985	25,538	0,598	-0,913	-2,966	1,140	0,536
Streptococcus	sum	-20,904	-56,290	14,483	0,251	0,301	-0,583	1,184	0,507
